# Students’ Development of a Logarithm Function in Python Using Taylor Expansions: a Teaching Design Case Study

**DOI:** 10.1007/s40751-022-00104-3

**Published:** 2022-04-26

**Authors:** Odd Petter Sand, Elise Lockwood, Marcos D. Caballero, Knut Mørken

**Affiliations:** 1 Centre for Computing in Science Education (CCSE), University of Oslo, N-0316 Oslo, Norway; 2 Department of Mathematics, Oregon State University, Corvallis, OR 97331 USA; 3 Department of Physics and Astronomy, Department of Computational Mathematics, Science and Engineering, and CREATE for STEM Institute, Michigan State University, East Lansing, MI 48823 USA

**Keywords:** Backward design, Computing, Mathematics, Taylor polynomials, Understanding

## Abstract

We present here the lessons learned by iteratively designing a tutorial for first-year university students using computer programming to work with mathematical models. Alternating between design and implementation, we used video-taped task interviews and classroom observations to ensure that the design promoted student understanding. The final version of the tutorial we present here has students make their own logarithm function from scratch, using Taylor polynomials. To ensure that the resulting function is accurate and reasonably fast, the students had to understand and apply concepts both from computing and from mathematics. We identify three categories of such concepts and identify three design features that students attended to when demonstrating such understanding. Additionally, we describe four important take-aways from a teaching design point of view that resulted from this iterative design process.

The intersection of computing and mathematics is a pillar of modern science, but there is relatively little research on integrating these two domains in teaching situations. In particular, it is rare that researchers identify and formulate teaching design principles for this purpose, and research should therefore support designing for understanding in mathematical–computational settings (Lockwood & Mørken, 2021). An interesting parallel is the intersection between mathematics and physics, where researchers have identified student difficulties and connected these to design principles that address them (see Caballero et al., 2013). The concept of Taylor polynomials exemplified there is also central to this article.

Our aim with this article is to showcase the design and implementation of a tutorial that integrates computing with mathematics to strengthen students’ understanding of important concepts in both domains. In presenting this tutorial, we will articulate important lessons we learned for instructional design. In particular, we will demonstrate that re-creating how the computer performs familiar tasks (such as calculating logarithms) can provide rich opportunities for students to make use of mathematics and computing knowledge in an integrated way, as exemplified by one of the key tasks of this tutorial: applying logarithm rules to the representation of real numbers in the computer’s memory.

For most students, asking an electronic device for the logarithm of a number can be characterised as a *black-box* operation, a term we have borrowed from computer science education (du Bolay et al., 1981): the number is returned as if by magic, with no reference to the means or processes underlying its calculation, nor any measures of its accuracy. While students depend on the correctness of these calculations, there is often little understanding involved beyond figuring out which buttons to press (Gravemeijer et al., 2017; Watters & Watters, 2006).

In our tutorial, we offered our students an opportunity to do the authentic work of programming their own logarithmic function using Python and, at the same time, to learn more about the usefulness of Taylor expansions. Doing so offered students an opportunity to reason about the mechanisms and processes behind the calculation of a logarithm, thus deepening their understanding of an important idea at the intersection of mathematics and computing — namely, how real numbers are represented and approximated in a computer.

Our work thus builds upon a rich history of existing literature that has explored teaching and learning at the intersection of mathematics and computing, including examining ways in which the computer and programming may reflexively support students’ mathematical thinking and learning (e.g. Benton et al., 2017, 2018; DeJarnette, 2019; Hoyles & Noss, 2015; Feurzeig & Papert, 2011; Papert, 1980/1993; Sinclair & Patterson, 2018). The results of these works are accounts of students’ and mathematicians’ experiences with mathematical computing, accounts of computational thinking in mathematics, and the development of learning environments and tasks. We particularly aim to extend previous work that has examined computing in university mathematics settings (e.g. Buteau et al., 2020; Buteau & Muller, 2017; Cetin & Dubinksy, 2017; Lockwood & De Chenne, 2020, 2021).

Lockwood and Mørken (2021) call for more studies on computing in mathematics education at this post-secondary level, particularly seeking additional examples of this integration in new contexts, domains, and topics. Our study answers this call by presenting a case of undergraduate students in Norway, using Python to reason about topics that include logarithms, Taylor expansions, and representing numbers in the computer.

This tutorial approach for addressing topics that students find difficult in an existing course is similar to that of Ambrose (2004) in introductory physics. The idea is not that students should be left to learn on their own without instruction, but rather that the tutorial guides their inquiry, provides feedback, and raises important questions, which they may then discuss with instructors and fellow students. Thus, we aim to avoid common pitfalls of minimal instruction, such as cognitive load, the worked-example effect, and not providing students with process worksheets to organise their work (Kirschner et al., 2006).

We based our tutorials on the framework of Wiggins and McTighe (2005), which is a three-stage process of *backwards design*: first, one attains clarity of the learning goals and defines the understanding that students should come to. Second, one determines what would be acceptable evidence for this understanding having taken place, and designs assessments to uncover that evidence. Finally, one designs the learning activities by which the students will be able to uncover the desired understandings.

We elaborate our research questions after defining some key terms and constructs in the following section. After this, we describe our methodology, after which the next five sections alternate between detailing the three stages of design and chronologically presenting our results from two testing (implementation) phases. Finally, we discuss our results and implications for teaching and future research.

## Relevant Literature and Theoretical Framework

In this section, we first review the *Understanding by Design* (UbD) framework that we used in more detail, and then elaborate on what black-box thinking means in this context. Finally, we present a literature review of how computing has been integrated into mathematics education and situate our work in the literature before presenting our research questions.

### Understanding by Design

In the Understanding by Design framework (Wiggins & McTighe, 2005), an *understanding* is defined as a specific and useful generalisation that points to transferable big ideas. Such understanding requires *uncovering*, making understood concepts as a result of inquiry and argument, as opposed to mere drill. We follow Lobato (2012) in defining the concept of *transfer* to mean any generalisation students make, without focusing on normative correctness. Wiggins and McTighe (2005) echo this sentiment in their discussion of assessment validity: “we typically pay too much attention to *correctness* [in our assessments], and too little attention to the *degree* of understanding” (p. 183; *italics in original*). In other words, we often mistake poor performance with poor understanding. Understanding differs from knowledge, but is also connected to it: “An understanding is a mental construct, an abstraction made by the human mind to *make sense of* many distinct pieces of knowledge” (Wiggins & McTighe, 2005, p. 37). In other words, pieces of knowledge are the dots that are connected by the act of sense-making to form an understanding.

According to Wiggins and McTighe, there are six kinds of understanding: being able (a) to *explain* general ideas, (b) to *interpret* specific instances of such ideas, (c) to *apply* the ideas and knowing when and how to use them, (d) to gain distance to the subject matter and see it from different *perspectives*, (e) to have *empathy* with ideas that seem odd or foreign at first glance, and (f) to have *self-knowledge* so as to know what one knows, what one does not know and how one’s learning is progressing (for more details, see pp. 82–104).

In the first phase of backwards design, where learning goals are in focus, it is important to prioritise. From least to most important, the curriculum is divided into knowledge that (a) is worth being familiar with, (b) is important to know and do, and (c) consists of the big ideas and enduring understandings that everything else hinges on (p. 71). For the latter, especially, a set of *essential questions* may be a useful tool for the teaching designer. These are not answerable in finality with a brief sentence, but are meant to have students ponder them and, in so doing, uncover desirable understandings. In short, an understanding makes use of facts, but is not a simple fact itself.

The second design phase focuses on evidence for understanding and assessment, and here, it is crucial to distinguish internalised flexible ideas from borrowed expert opinions that students have memorised as facts to be delivered on cue. This may sound like understanding, but students will often fail to recognise that these ideas are relevant in novel situations or fail to adapt the ideas when the situation requires it (see pp. 46–50). To make this distinction necessarily involves supplementing the traditional quiz or test with academic prompts and performance tasks*. Academic prompts*, of which our tutorials are examples, pose questions or problems that require critical thinking, explanations, and defence of the answer and methods. *Performance tasks*, on the other hand, ask students to do authentic work that yield tangible products and performances, and give students opportunities to personalise the task.

Following Wiggins and McTighe, for us to claim that the students understand, they need to provide reasons and support for their choices, in line with the six facets of understanding. It is important that the students’ answers are not dependent on blatant cues. In our study, therefore, we have used the framework to label an event as “understanding” when students explain, interpret, apply, or demonstrate perspective without regurgitating statements from the tutorial or the interviewer. On some occasions, vague hints were necessary, but when the students interpreted those hints correctly, explained why the information was relevant, or applied the hint in a constructive way, it still gets classified as understanding in these terms.

Finally, the third phase of backwards design places the focus on learning activities, of which direct instruction (teaching) is but one example. The optimal designs provide students with engaging and effective tasks. An *engaging* task is recognised as meaningful and intellectually compelling by the learners and presents them with a mystery or challenge with which they can go hands-on. *Effective* tasks are ones that help learners become more competent. The goals of such tasks are clear, the criteria are well known, and the students are given opportunities for self-assessing along the way.

In the work of Wiggins and McTighe (pp. 197–224), a set of design prompts is suggested for analysing the learning activities. These implore the designer to ask whether one has made it clear to the students *where* the unit is headed (and *why*), to *hook* (and *hold*) their attention, to allow them to *experience* doing the subject, to *rethink* (and *reflect*) along the way, to *evaluate* their strategies, to *tailor* and personalise the task to their own preferences, and to *organise* the activity using a whole-part-whole^1^ format. The italicised words’ first letters spell out WHERETO, which is the name given to these design prompts.

### Black-Box Thinking

While not formally a part of the UbD framework, as noted previously, the term *black-box thinking* could be applied to situations where a student uses results without understanding the underlying process. Falbel (1991) argued that tools, such as computers, should be transparent and *convivial*, in the sense that the social arrangements people create around their use should afford the users of these tools to invest the world with meaning:

When home computers started to appear in the mid-1970s, they were […] bought in kit form to be assembled at home [and] were designed to be tinkered with. Not so any more. […] This constitutes a reduction in conviviality, the hallmark of which is self-reliance. The computer is becoming more and more opaque—a veritable black box. The message here [on the label] is clear: You cannot understand it; you must rely on the experts. […] Learning to program a computer can in some sense make it a more convivial tool [and] allows one to shape the tool to one’s needs and tastes. This allows for more freedom and flexibility as far as what the computer can be made to do and what it can be used for. (pp. 32–34)

Importantly, when students engage in black-box thinking, we should be careful not to say that they are *unable* to understand, but rather that they have not engaged with how the result was found as something to be understood. In these cases, it may simply be that the task does not require students to attend to this aspect: all that is asked of them is that they get a correct answer without highlighting the importance of explaining how that answer was derived.

Thus, while black-box thinking can be said to represent knowledge, in the sense that the students know how to formulate a query of the computer to get an answer, understanding in our context means that they also know *how* the computer finds the answer, and that they are able to interpret and connect it to other forms of knowledge as well. In our interpretation, black-box thinking is not what Wiggins and McTighe (2005) would consider to be understanding, but it is nonetheless particularly relevant for contexts that involve computing.

### Literature on Integrated Design

Integrating mathematics learning with computing is perhaps best illustrated by a counter-example. In what we might call a *dis*integrated design, the students learn to code in a context that is mathematical only by accident, with learning activities such as creating a web-page for flight booking with a database back-end. Presupposing, then, that students know how to program, instructors introduce them to the relevant math software libraries and focus on the *application* of these presupposed skills.

A potential problematic issue with this approach is related to *transfer* of learning: the context in which learning takes places has an impact both on the learning itself and on the potential for transfer to other contexts (Billett, 2013). Hence, learning to code in a non-mathematical context plus learning mathematics does not necessarily imply that students will be able use code effectively *in* mathematics. However, as computational methods are becoming part of the scientific disciplines to an increasing degree (Weintrop et al., 2016), opportunities now exist to have students do authentic scientific work that involves computing.

Indeed, science education researchers have explored the integration of computational thinking and practices within science contexts among school aged children (e.g. Farris & Sengupta, 2014; Farris et al., 2020; Sengupta et al., 2018; Wagh et al., 2017) and undergraduates (e.g. Caballero, 2015; Caballero et al., 2019; Hambrusch et al., 2009; Magana et al., 2013; Odden et al., 2019). Such work highlights endeavours in the research community to better understand ways in which computing might support science education.

In the mathematics education literature specifically, there are numerous examples of different computational tools being implemented as part of learning activity designs in university mathematics. For instance, Dimiceli et al. (2010) showcase a design where the symbolic Computer Algebra System (CAS) features of the *WolframAlpha* app are being used as an asset in an introductory calculus course. In another instance, Caglayan (2016) demonstrates a way to use the *GeoGebra* dynamic software to visualise Riemann sums in a similar setting. Beyond showcasing designs that incorporate these technological tools, there are also studies that investigate the relationship between task design and students’ use of them. Olsson (2019) comparatively investigated two designs that used *GeoGebra*, in this case a task involving functions designed for schoolchildren in grades 7 to 9.

There are also examples of software being designed specifically for educational purposes. One such example is *Grid Algebra* (Hewitt, 2016), a software designed for learners as young as 9–10 years old to visualise the four basic arithmetic operations as movements on a grid when solving linear equations. The software called *Configure* (Greenstein, 2018) similarly lets younger students visualise and conceptualise topological equivalence. In their respective articles, both of these authors outline their strategies and principles that they employed in designing their software.

In addition to using pre-existing software and writing dedicated software for educational purposes, there is a third option: having students write or modify computer programs written in a generic programming language. An integrated approach then demands that these programs are written in a mathematical context. Examples of this include the work of Lockwood and De Chenne (2020), in which students relate combinatorial counting problems of different types to corresponding conditional statements in Python programs, and that of Ramler and Chapman (2011), where students statistically analyse whether missed notes in the *Guitar Hero* video game were randomly distributed by writing code in R.

The work we present here resembles these last two examples and belongs in the same category. Our work is focused on university students using the Python programming language in a mathematical setting (the context is described more closely in the first sub-section of the next section). In this article, when we say *computing*, we refer to *machine-based computing*, “the practice of developing and precisely articulating algorithms that may be run on a machine” (Lockwood & Mørken, 2021, p. 405). In practice, that means our students used Python programming to articulate, visualise and solve mathematical problems.

Buteau and colleagues (2020) point to a central feature of what this mathematical coding entails: in order to articulate a mathematical process in a programming language, one translates into the language what one would do by hand. To do this, one must realise that the code can indeed work in a similar manner as one does by hand, which is neither self-evident nor independent of what kind of mathematical work one engages in. Integrating coding in mathematics then entails supporting the students in learning: in the words of Buteau and colleagues, “to transform a programming technology into a rich ‘mathematical instrument’ enabling him/her to [participate] in programming-based mathematical work” (p. 1029).

### Research Questions

Having described understanding by design and articulated our attention toward integrated design, we now present our research questions. When using the term “understanding” in these questions, we mean specifically the characterisation of understanding given by Wiggins and McTighe (2005) previously discussed.

1. Which features of the tutorial design do students attend to when they demonstrate understanding of a mathematical concept (as opposed to black-box thinking)?
2. Of which mathematical and computational concepts did our students demonstrate understanding?
3. What did we, as designers, learn about designing for students’ understanding from the iterative process of tutorial design?

## Methodology

We classify this study as an instance of *design-based research* (DBR), where information about students’ learning experiences informs subsequent rounds of design and instruction. In DBR, one defines pedagogical outcomes and creates learning environments that address them. One modifies the process until the desired outcomes are attained and one finally reflects on the process. A strength of DBR is that it allows researchers to address complex, often cross-disciplinary, problems. In addition, DBR also lends itself to authentic, inquiry-based tasks, is able to reveal new design principles, and works on long-time scales (2 to 5 years) with continual refinement (Reeves et al., 2005).

One limitation of DBR in the form of case studies, as we use here, is a lack of generalisability. Therefore, the research questions just presented should not be interpreted as aiming for a theory that is universally valid. There is also a concern that such studies are biased, in the sense that they involve the researcher’s subjectivity. This concern can be alleviated to some extent by triangulation of data from multiple sources and cross-validation by other researchers (Teegavarapu et al., 2008). We collected video, code, worksheets, and asked follow-up questions of our subjects, and the other authors helped triangulate the propositions of the first author (see the third sub-section below).

We find all of this to be in alignment with the design principles of Wiggins and McTighe and our research questions above. To identify design features that students attended to and described the concepts they understood, we needed several cycles of design to produce the desired outcomes. In particular, the final research question, with its focus on extracting design principles from our experience, aligns well with DBR.

### Context of Study

A *tutorial* is defined as an educational approach where, “instructors are provided with a classroom-ready tool to target a specific concept, elicit and confront tenacious student misconceptions, create learning opportunities, and provide formative feedback to students” (NRC, 2012, p. 129). Besides the research itself, the tutorials we made were important end-products of the process. It was important, however, that the research helped define the standards and contributed to the quality of these products.

We designed three tutorials for the first-semester course MAT-INF1100: “Modelling and computations” (Mørken, 2021), which is in common to mathematics, physics and electronics students at the University of Oslo (UiO). This course is taught alongside courses in calculus and programming, and is intended to link these courses together. This set of courses is intentionally co-ordinated. Typically, a mathematical concept is first covered in calculus; then, MAT-INF1100 covers how to implement the concept numerically, and finally, the students write Python code to do just that in the programming class, as described by Malthe-Sørenssen and colleagues (2015).

Additionally, a few other models for such integration exist, for example, co-ordinating joint computational projects for mathematics and engineering courses at Chalmers University of Technology, Sweden (Enelund & Larsson, 2006; Enelund et al., 2011). Some universities offer explicitly designed courses to foster, such as MICA (Mathematics Integrated with Computers and Applications) at Brock University, Canada (Ben-El-Mechaiekh et al., 2007; Buteau & Muller, 2017). Furthermore, occasionally mathematics and computing are integrated in a third context, like bioscience (Nederbragt, 2020).

However, the UiO model, in which concepts are reinforced across multiple courses and programming is systematically integrated for first-year students, is not common. We point this out to provide some overall context for the study we describe, and to situate our design activity within the broader departmental, programmatic, and university systems in which our study took place.

### Data Collection

**Fig. 1:**
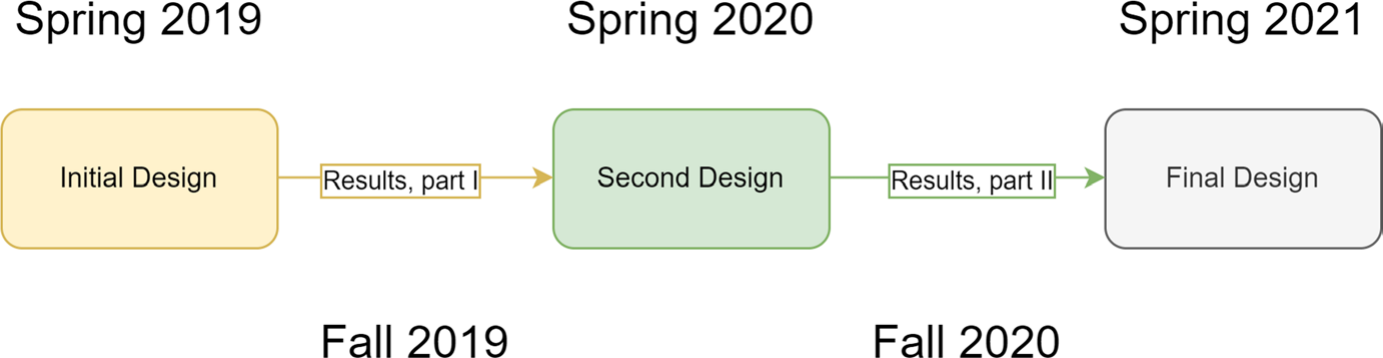
Phases of the design experiment with corresponding subsequent sections of this article (the boxes indicate the three design phases, while the arrows indicate the two implementation phases, with data collection and analysis)

The study was designed and conducted in five distinct phases, as shown in Fig. 1:

For the two implementation phases, we recruited students for interviews in two ways: (a) asking them to volunteer using an online form during one of the first lectures of the semester and (b) recruiting groups of students during in-class observations (not possible in the final interview phase due to COVID-19 requirements).

In the initial implementation phase, we conducted eight interviews with a total of thirteen students, of whom five participated in two interviews and the rest in one. Each interview covered just one of the three tutorials we designed. Gina and Benjamin in the Initial Design phase had already been interviewed using an earlier tutorial, whereas Martin, Lydia, and Roger from the same setting would be interviewed again using a later one.

In the second implementation phase, we conducted seven interviews with seven students, of whom four participated in three interviews (all the tutorials) and the rest in one. Rita and Lena from the Second Design phase were present in all three interviews, and the interview we present excerpts from here was their second one.

In both phases, the first author interviewed the students and gave them instructions to work together to solve the tasks as they would in class. We captured a video of the students and the whiteboard they used, a separate video of their work on the computer screen, and an audio recording of their conversation.

The students were told to work together as they would have in a normal group session for the course, using the interviewer as a teaching assistant to answer questions. If the students did not ask, the interviewer would initially refrain from intervening, in order to have as much data as possible be attributed to the tutorial designs and not subsequent interventions. However, if there was danger of the students spending too much time progressing down the wrong path (or not at all), the interviewer would intervene once we had a clear picture of what the students intended to do. This was done to ensure they would have sufficient time to complete the entire tutorial.^2^

Follow-up questions were saved for the end of the interviews: specific questions asking students to elaborate on interesting choices and actions during the interview were noted down by the interviewer as the interview progressed and asked about at the end. While this tends to affect validity, as students have to recall their experiences from earlier in the interview, it ensures that their thought processes during the interviews themselves were affected as little as possible by the interviewer (van Someren et al., 1994). More general questions pertaining to the students’ backgrounds and their thoughts on the tutorial designs were asked at the same time. These questions were agreed upon by the authors ahead of time.

### Analysis

To analyse the data, we employed the pre-existing framework of Wiggins and McTighe (2005) in a top–down approach^3^ to identify evidence of understanding. After the interviews were concluded, we had the audio transcribed and the first author flagged episodes that matched the evidence for understanding that was identified during the preceding design phase.

This evidence corresponded to the six facets of understanding. We rarely saw students express empathy with outside points of view or self-knowledge, and we suspect the tutorial did not promote these forms of understanding to any significant extent. In many instances, however, our students did explain, interpret, apply knowledge, and demonstrate perspectives.

As discussed earlier, the way we used the six facets of understanding required students doing these things without blatant cues from the interviewer. This is not to say that no understanding could result from hints (which would be made intentionally vague so as not to rob the students of an opportunity to demonstrate understanding). Ideally, the students would also justify the choices that they made, but in some cases, we relaxed this requirement. For instance, when Rita and Lena made use of mathematics to solve what we, as designers, imagined would be a computational problem, and then used the mathematical results to simplify their computer program, we labelled this a case of applying knowledge, even though the students did not justify the latter action explicitly. This should be considered a methodological limitation, and the remedy, from a future research point of view, is to include follow-up questions that address this specifically.

The first author translated the episodes from Norwegian into English and enhanced them by inserting images from the video recordings to provide additional insight into how the students worked with the tutorial. The first author then coded the flagged episodes according to which facets of understanding the students exhibited, and what they paid attention to at the time or just before, as evidenced by what they looked at (in the video), worked with or talked about. The rest of the research team independently reviewed and validated these coded episodes. It is possible that the students could have paid attention to or thought about things we were unable to capture with this approach, which is an important limitation. We therefore do not claim to have found the complete set of features and understanding.

We also checked the tutorial text to ensure that the understandings were the students’ own: for example, when students expressed an understanding that mirrored text from the tutorial or hand-out, these episodes were omitted to ensure that we did not mistake a borrowed expert opinion for the students’ own. We then examined the *content* of these understandings (*of* what the students demonstrated understanding).

Finally, the first author selected the episodes that most clearly impacted the design process or otherwise provided evidence of the tutorials working especially well or poorly according to the learning goals. We partly based these justifications on follow-up questions to the students after each interview, which helped inform the subsequent design phase. Once again, the other authors validated this selection. Even though this article is an exploratory case study, we wanted to ensure that we picked representative cases that each added something to the take-aways we formulate at the end of the article, including cases that we, as teaching designers, found illuminating and instructive in the process.

In the following section, we describe the evolution of the Taylor expansion tutorial through each of the five phases described in the previous sub-section.

## Initial Design: Choices in Taylor Expansions

The initial design consisted of the three phases of backwards design: learning goals, evidence for understanding, and learning activities.

### Learning Goals

The learning goals for the initial design sought a compromise between several concerns. Firstly, we considered the potential for including a significant computational component that warranted a hands-on approach. Some of the pre-existing course material tended to lean toward the mathematically abstract side of things, and we wanted to bring in the computational domain to a larger extent than before. Secondly, we did not want to stray too far from the pre-existing learning goals of the course (as summarised in Mørken, 2021), so as not to risk the students experiencing the activity as irrelevant. Thirdly, we wanted to follow Wiggins and McTighe’s (2005) definitions of an understanding as something that needs to be *uncovered*, not covered; something that makes use of the facts, but also that explicitly requires the learner to make sense of the content. Finally, we wanted to focus on a topic which students had difficulty from the traditional approach, in the experience of faculty teaching the course.

We reviewed the learning goals at the course level and discussed the focus of the tutorial with faculty teaching the course. They suggested that one topic that students found particularly difficult was the fact that certain functions would display divergent behaviour in certain regions when adding additional terms to a Taylor series: the more terms one adds, the worse the accuracy gets. We found this counter-intuitive behaviour to be an excellent example of a concept that required uncovering. The reasons for this behaviour are detailed in Appendix 1.

Our learning goals for the initial design were thus that the students should understand that:

- more terms are not always better;
- the choice of the point $a$ around which to construct the Taylor expansion is not always merely a matter of convenience;
- while we can make the mathematical error (remainder) arbitrarily small, the presence of rounding errors means that there is a practical limit to how good the approximation can become on a computer.

### Evidence for Understanding

Having defined the learning goals, we moved on to examine what we would consider credible evidence for students understanding the concepts involved. An important limitation of our context is that we were not re-designing an entire course, but rather just designing tutorials to fit into one. Throughout the semester, in each of the three weeks when a tutorial would be run, we had access to 1 hour of the students’ time in class and up to 2 hours for the students who participated in interviews. Therefore, we were not in a position to design formal assessments; whatever evidence we required needed to be part of the tutorial itself.

To that end, we designed the tutorials as academic prompts. These required the students to think critically and not just recall knowledge. The focus was on students providing explanations and defending their choice of methods. Typically, such problems are open-ended, which was not the case here: there existed an optimal choice for the pair of parameters $a$ and $n$ (number of terms in the Taylor expansion).

Even so, by requiring that the students use their hands-on experience with the computer program to justify their eventual choice of parameters, we expected that the tutorial worksheets would be able to provide evidence for four of the six facets of understanding as follows: (a) explain (and prove) why the choice of parameters is important, (b) interpret the plots (identifying rounding errors), (c) apply their knowledge by identifying optimal parameters and writing code that measured the error, and (d) display self-knowledge by comparing their initial intuition with experiences from working with the tutorial and reflecting on the process.

### Learning Activities

The initial tutorial design took a cue from the Maryland Tutorials in physics (Redish, 2009), by having students use their intuition to sort out statements about Taylor polynomials into those with which they agreed and those with which they disagreed. Some of these would be statements that often, but not always, apply, such as, “adding more terms increases the accuracy of a Taylor approximation.” This task sought to have students think about what they believed to be true regarding Taylor polynomials, and we repeated the same task at the end of the worksheet for comparison. In this repeated task, students were given the opportunity to comment on statements of which they had changed their opinion, or statements which they interpreted differently after having completed the tutorial.

After the initial task, we asked students to find expressions for the Taylor expansion of $f\left(x\right)={x}^{-2}$ and its remainder. We provided them with code that plotted the remainder for various choices of the parameters and asked them to look for patterns in these plots (see Appendix 1 for details).

Next, we asked them to analyse their own formula for the Taylor expansion and identify the factor that would eventually dominate, and then determine for which parameter choices this factor would grow without bounds. This factor would be the main source of the divergent behaviour and identifying it would also help students identify the region of stability where the expansion always converges.

Their next task was to compare the result with the plots and explain how they were connected (the students would be able to see the region of convergence in the plots). Finally, we asked them to explain what it means for a Taylor polynomial to converge. The idea was that they would connect the known concept of convergence for a series of numbers to convergence of Taylor terms in the sum. As the final step of the design, we used the design standards of Wiggins and McTighe (2005) to validate the learning goals, evidence of understanding, and learning activities.

In the following section, we will convey the results of this first design, followed by an account of the next round of design in the following section. The result of that design then follows. We describe our final design in the penultimate section, followed by a discussion of the results and design lessons in the final one.

## Results, Part I: Initial Implementation

In this section, we describe some results from the first round of implementation. We present data from two different interviews — one with four students and one with three — and describe what we learned from those interviews. In particular, we focus on where the tutorial described in the previous section fell short of our expectations, and the changes we made based on these experiences. Most of the relevant excerpts from the interviews will be summarised.

Once the design was finalised, we set up interviews with volunteer students recruited from earlier in-class observations of an earlier tutorial, as well as some students who had volunteered to be interviewed at the start of the semester. Due to scheduling conflicts, we had to break up the groups, so that some, but not all, of the students had worked together before.

The interviews for each tutorial were scheduled the week before that tutorial was to be used in class. This is likely to have raised the difficulty for the interview groups, as some students might not yet have been familiar with Taylor expansions.

### Benjamin, Gina, Martin, and Ruth

The first group consisted of four students. Martin had had previous experience with computer programming and often suggested what the group should do. Gina was verbally active and asked many questions throughout the interview, often initiating discussion around the mathematical concepts involved. Benjamin and Ruth spoke less than the other two and, for the most part, contributed observations and questions without guiding the activity of the group as much as Gina and Martin.

Martin solved the initial mathematical task to find the Taylor expansion almost singlehandedly, as he was the only student who was familiar with the concept. The others expressed that they had not had time to look at the material by the time of the interview. Gina took charge of writing the Python code that reflected Martin’s work on the whiteboard. With input from the rest of the group, she wrote the necessary functions and ran the code to produce plots.

As it happened, Martin made a mistake that made it difficult to reproduce the expected results: in the Taylor expansion, he differentiated with respect to the wrong variable, ${f}^{\left(i\right)}(x)$ instead of ${f}^{\left(i\right)}(a)$. We conjecture that this happened because the tutorial used that variable in the example derivatives: ${f}^{^{\prime}}\left(x\right)=-2{x}^{-3}$ instead of ${f}^{^{\prime}}\left(a\right)=-2{a}^{-3}$. When this happened, Martin was attending to his correct formula for the general Taylor expansion on the whiteboard, and his general formula for the *derivative* (using the inappropriate variable) on his worksheet.

**Fig. 2:**
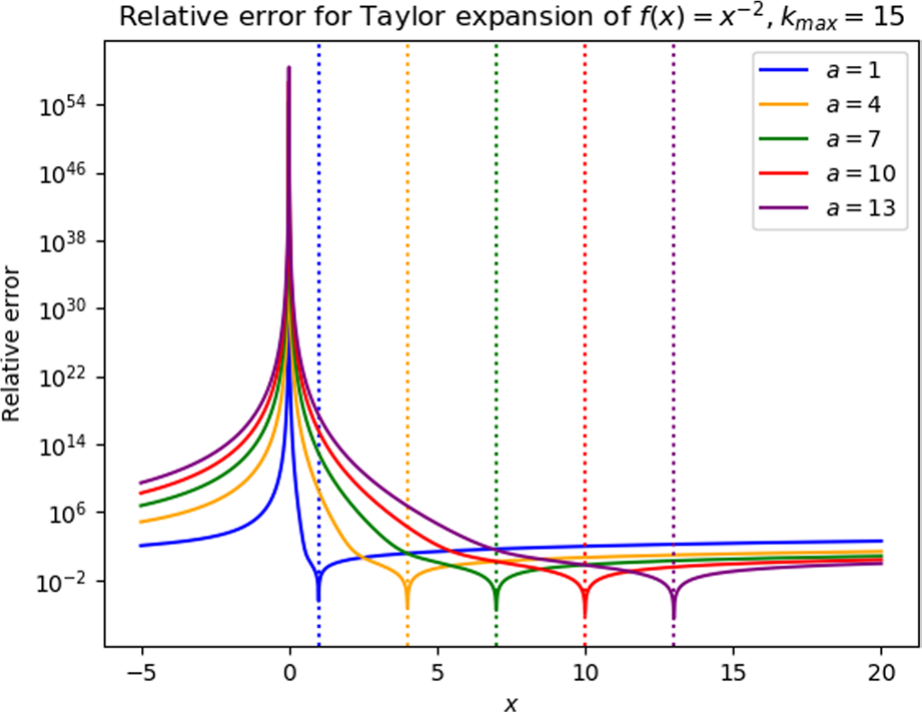
Plots produced by the students’ code in the first-phase interview (note the difference with Fig. 11 in Appendix 1)

He displayed understanding in deriving these general formulas and was able to explain what he was doing to the other students. The result was a series of plots that looked almost right, but not quite. In interpreting the tasks, students assumed their plots (such as the one in Fig. 2) were correct, when they did in fact look quite different from the expected results (as shown in Fig. 11 in Appendix 1):

As a result of this, the interviewer had to intervene and alert the students to the fact that their plots did not look right. A cursory examination of the code did not reveal the error, which was only discovered by the first author after the interview had concluded. In the end, the interviewer had to show the student figures of correct plots that they could use to answer the questions on the tutorial worksheet. As such, the tutorial did not succeed in giving the students opportunities to self-assess, as they had to depend on the interviewer for this.

Another problem that required interviewer intervention was the task asking students to analyse which factor in the Taylor terms would dominate when the number of terms grew large. As it turned out, the expression Martin wrote on the whiteboard^4^ for Taylor term number $i$

$$T\left(x,a,i\right)=\left(i+1\right){\left(-x\right)}^{-2-i}{\left(x-a\right)}^{i}$$

did not group all the powers of $i$ together as we had anticipated, but instead kept them as two separate factors. As a result, it was difficult for the students to pick any one of these factors as the dominant one — it would depend on how far $x$ was from $a$. Again, it was demanding for the students to self-assess their expression. They were able to interpret what was asked of them and to explain their thinking, but the tutorial did not support them in figuring out what do to when they did not know what to do, to put it in the terms of Wiggins and McTighe (2005).

The interviewer again had to intervene, assuring the students that they could not have known that writing the expression in the way they did would make the task difficult to complete. Gina then transformed Martin’s expression into a more usable one on the whiteboard. It turned out that even with an expression that was easier to work with, the students had some difficulty seeing that ${c}^{i}$ would eventually grow larger than $i$ for any $c>1$.

In particular, the interviewer’s example of ${1.01}^{i}$ revealed that they seemed to focus on the slow initial growth of ${1.01}^{i}$ compared with $i$ itself. This seems to have prevented them from seeing (or remembering) that exponential growth will always overtake linear growth at some point.^5^ Only when the interviewer pointed this out to them did they agree that $1$ was the limit for the exponential term dominating the growth, and that ${0.99}^{i}$ would drop off toward zero as $i$ grew larger. This blatant feeding of information unfortunately left little for the students to uncover.

While the students in this instance were able to justify their answer and generalise knowledge about functions, it also demonstrates that this version of the tutorial often got the students caught up in details rather than pointing them toward the big ideas and essential questions.

In terms of the design principles, it was not clear to them where the instructional unit was headed and why, which would have been necessary for us to claim that the tutorial was effective. This was amply illustrated toward the end of the interview, while asking follow-up questions:

*Interviewer: *Have you thought about why we are calculating approximate functions that we already have exact expressions for? What is the point of that? We do have …

*Gina: *I’m wondering the same thing, I don’t really know, ha-ha. I was thinking …

*Benjamin: *But what is it you use Taylor polynomials for, really?

*Interviewer: *Didn’t [the professor] tell you?

*Benjamin: *I didn’t catch it, but I think [inaudible]

*Ruth:* [inaudible] didn’t say that much about it either

*Martin: *Erm, as long as you don’t take a limit where [the number of terms] goes to infinity then you have a, is it closed form it’s called? Like, one that you can calculate simply, though. […] A sine [inaudible] has a direct one, or like an expression you can just plug everything into.

*Interviewer: *[Crikey], that reminds me of something, because if there was no log function in Python, how would you be supposed to calculate it?

*Martin: *Yes. [Gina and Ruth voice agreement]

*Interviewer: *Then you could actually, ah, I have no idea how the logarithm is calculated. But you could use Taylor, but then you just have to know about the limitation,^6^ heh-heh.

*Martin: *But I think it’s that way the computer does it with the [inaudible] functions, that they make some approximation or other of the type Taylor polynomials or something, and uses that to calculate …

*Interviewer: *Yeah.

*Gina: *Huh.

*Ruth: *Cool.

The students indicated that they would be interested in trying to make their own log function using Taylor polynomials. This would have provided a means to hook and hold their attention that was missing in this first version of the tutorial. As such, the tutorial was not as engaging as we would have hoped. Unfortunate as these problematic issues were, the initial implementation did give us opportunities to adjust and improve the tutorial.

In sum, this interview alerted us to four issues with the tutorial: (a) we misled the students into differentiating with respect to the wrong variable; (b) we assumed the students’ Taylor expansion to be of a form that easily would allow them to identify the dominating factor, which was not always the case; (c) we assumed the students were used to reasoning around identifying with dominating factors, which they were not; and (d) we lacked a way to hook and hold the students’ attention.

### Roger, Mathias, and Lydia

Before we move on to the next design phase, however, we summarise an excerpt from another interview, which took place later the same week. We had been able quickly to tweak some of the issues that arose for the first group of students; namely, we updated the example to use the variable the students would need to use, and we provided a plot that students could use to check their results against. Note that these were only minor edits and did not constitute a new design phase.

In this interview, three students (whom we call Roger, Mathias, and Lydia) were working on a slightly revised version of the same tutorial. Mathias filled much the same role as Martin in the previous group, having had previous experience with Python programming as well as being able to explain mathematical concepts to the other students. Lydia, like Gina, was verbally active and used questions to drive the process of understanding the assignment. Roger, generally, was more focused on completing the assignment as a goal of its own and participated in the discussions as a means to that end.

Thanks to the aforementioned revisions, this group of students did avoid differentiating with respect to the wrong variable and obtained the correct plots. But Roger several times expressed a concern about them having trouble seeing the forest for the trees, as it were. A little later, he elaborated on this when prompted by the interviewer, connecting his confusion to his lack of understanding of Taylor polynomials as a concept at that point in the semester. Lydia, on the other hand, seemed to have difficulty keeping track of all the different variables involved.

The second interview thus alerted us to the following issue: the tutorial’s focus was too narrow and did not point the students towards resolving the issue that was essential from their point of view (What *is* a Taylor polynomial? What is it good for?). Furthermore, the amount of information the students had to attend to felt overwhelming at times.

In both interviews, the tutorial overshot the 1-hour target and, as a result of these issues, it was dropped from a wider test run in class the following week and flagged for a thorough redesign from the ground up. In spite of these very real problems, the tutorial did succeed in one thing: the students spent a lot of time trying to make sense of what they were doing and, in many instances, provided evidence for their understanding by explaining their reasoning. Even though the tutorial was successful in this regard, we still wanted to create a version of it that also gave the students a clear goal, made Taylor expansion seem relevant to them and did not overwhelm them with information. We also wanted to provide more opportunities for self-assessment, so that the students could have realised earlier and by themselves that something was off with their plots.

## Second Design: What Are Taylor Expansions Good For?

During and after the initial round of interviews, we discussed the emerging issues with course instructors and education research colleagues. We compiled the following list of improvements that could be made to the tutorial for the second design phase: (a) to forego the narrow focus on convergence of Taylor polynomials for the bigger issue demonstrating a use for approximating known functions, (b) to develop the tutorial to *show* students one way Taylor polynomials are useful, not just tell them, (c) to provide sufficient scaffolding so that students are not using all their cognitive resources to keep track of the different variables, and (d) as far as possible, to retain students’ engagement with the concepts and help them not use the computer as a black box.

### Learning Goals

An issue with Taylor polynomials that we identified in the first round of interviews is that students may not understand the point of them: if you already have the exact function, why bother with an approximation that is less accurate? Finding good motivations for Taylor expansions can also be a challenge for teachers (Johnson, 2011). Some even suggest that we might not want to motivate Taylor expansions at all (Šikić, 1990).

An often-overlooked point is that, in computers, approximations are central to computing most quantities that go beyond the four basic arithmetic operations. While these operations, that computers excel at, will suffice for calculating any polynomial, other common functions such as exponential, trigonometric, and logarithmic functions are not at all trivial for computers to deal with (Arlin, 2012). As such, we depend on these approximations every day, often without being aware of them.

From an educator’s perspective, we noticed that the two issues mentioned above are intricately connected. If we imagine, as we did in the interview quoted in the previous section, that we are re-inventing the computer from scratch, how would we go about programming the first logarithm function? The students in the interview responded with interest when confronted with this mystery. What *is* the secret behind this magician’s trick?

Taylor polynomials would seem to provide a solution, as polynomials only require the computer to master the four basic mathematical operations. But this simplicity comes at a heavy cost: one must pick a point $a$ that is close to the input value $x$ to create the polynomial; otherwise, the number of terms required for sufficient accuracy is prohibitive, even for a modern computer.

To make matters even worse, if the function is a logarithm, we have to pick the point that we expand around with care, or the accuracy may *worsen* as we add more terms (see Appendix 1). It seems that creating one Taylor expansion for all values of the logarithm would require us to expand around a point so far from 0 that the accuracy would be abysmal for small numbers. On the other hand, if a way could be found to overcome these difficulties, we would, in effect, have killed two birds with one stone: Taylor approximations would be useful in a fundamental sense and the way logarithms are calculated on the computer would become much more transparent to us.

As the second version of the tutorial neared completion, it was suggested by faculty teaching the course that we also include the concept of the remainder in the tutorial. The reasons for this were (a) that students struggled with this concept and could benefit from hands-on experience with it and (b) that if the students were making their own log functions using Taylor polynomials, the remainder would be useful to them as it provides an upper bound of the error.

We ended up with the following revised learning goals for the second version of the tutorial.

The students should be able to:

- explain how computers calculate logarithms;
- explain the usefulness of Taylor polynomials for known functions;
- explain how the representation of real numbers on the computer is helpful here;
- interpret plots of the remainder;
- apply what they have learned to pick good parameters;
- see the problem from both a mathematical and computational perspective, and be able to merge these perspectives;
- identify own preference for working computationally or mathematically and self-assess their understanding of either.

### Evidence for Understanding

To ensure that we had evidence for all these understandings, the updated tutorial explicitly asked the students to explain and interpret. A stated goal of the tutorial was that every student in the group should understand what was going on, and they were encouraged to discuss things they were unsure about with the teaching assistants. The final tasks invited the students to reflect on using mathematics and computing together in this fashion.

### Learning Activities

The new version of the tutorial first introduced the essential questions: how are logarithms calculated and what are Taylor polynomials good for? Then, the students were given functions that calculated the Taylor polynomial and were asked to write a function that calculated the absolute remainder. We listed and explained all the functions and variables involved, in order to help students not become overwhelmed by all the symbols, as Gina had been in the previous version.

The starting point for the re-designed tutorial was to figure out how computers actually calculate logarithms of real numbers. The key to acceptable accuracy turned out to be a mapping that reduces the range of the function from all positive real numbers to a very small region (see Fig. 4 below and Appendix 2). This elegant trick not only addressed both the first two bullet points listed above, but also additionally leveraged and made relevant something that was already part of the curriculum: understanding representations of real numbers on the computer.

After this was done, we incorporated the concept of the remainder, so that the students could control and predict the error of their approximation. We anticipated that such an approach could support the idea of re-inventing the logarithm from scratch, which our students in the first round of interviews found so intriguing: being able to estimate the error without needing to compare with a pre-existing function would add to the learning activity’s authenticity.

When the students had a working remainder function, we asked them to implement their own log function in several steps: (a) provided the representation of real numbers in the computer, find out what happens when you apply the logarithm to such a number; (b) implement the result as a function in Python using a Taylor expansion; and (c) self-assess their work against a test case (see Fig. 13 in Appendix 2). We gave the students a machine-accurate value of $\mathrm{ln}(2)$ to use in their function.

Next, the students combined the results of the previous two tasks. They could change the parameters in the remainder plot to find parameters that made the remainder accurate enough in the entire interval between 0.5 and 1. We found that an upper limit of ${10}^{-10}$ for the remainder made Taylor expansion accurate enough for our purposes. Then, they were to use these same parameters in their own log function and compare the result with the log function from the standard numpy library in Python.

Finally, we asked the students to reflect on the big ideas involved and their own learning.

## Results, Part II: Second Implementation

We conducted a new round of research interviews with students in 2020, one year after the initial round. This time, the interviews took place at the same time as the group sessions, where the other students worked on the same tutorial in class. From our experience with the first implementation phase, we deemed the designs to be finished enough that there would be no longer be a need to update them between interviews and classes. This change also helped with scheduling, as we knew the students to be available for interviews at that time and making sure we did not mix students from different cohorts.

Due to COVID-19 safeguards, we interviewed two groups of only two students for this particular tutorial and gave them dedicated tasks. One student was designated to work on the computer, which mirrored its screen on a large external display for the other student and the interviewer to see easily. The other student was designated to work on a large whiteboard when necessary. The interview protocol was otherwise unchanged from the first phase.

Most of the interesting episodes in the second phase came from our interview with two students whom we will call Lena and Rita. They were both fairly outspoken and took initiative to drive the work forward. Rita expressed a preference for working mathematically over doing programming and chose the whiteboard, while Lena, who worked the computer, had some limited coding experience from high school IT classes that mostly focused on web pages, databases and the like. Nonetheless, throughout the interview, they both contributed substantially to the work on both the whiteboard and the computer.

**Fig. 3:**
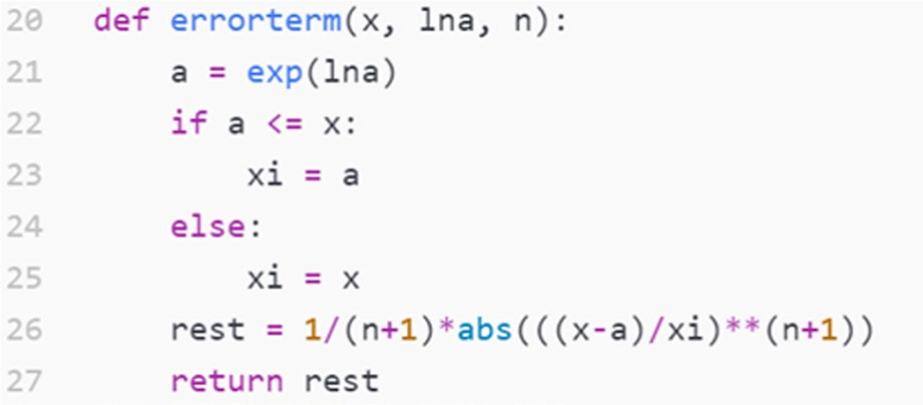
Rita and Lena’s implementation of the function that calculated the remainder(They wrote lines 22–27, while the rest were given in advance as scaffolding. The function name *errorterm* was, in later versions, changed to remainder, in order to evoke better the relevant mathematical concept in familiar terms.)

To save time, we provided Rita and Lena with a sheet that contained both the general remainder formula and the formula as applied to the logarithm specifically. Based on this, the students wrote the code in Fig. 3, which calculated the value of the remainder and then used provided test code to make a plot that confirmed the correctness of their implementation.

### Using Taylor Polynomials to Approximate the Natural Logarithm

**Fig. 4:**
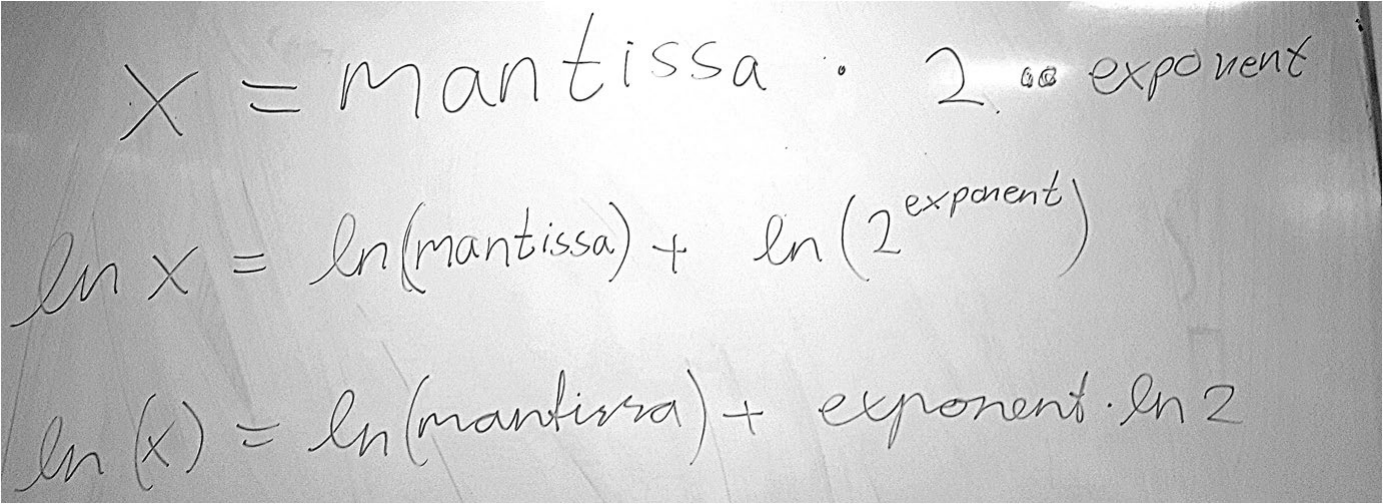
Rita’s usage of logarithm rules on the machine representation of $x$, effectively reducing the domain of the logarithm from all positive numbers to allowed values for the mantissa (between 0.5 and 1) (The variable names are the same ones we used in the tutorial text, intended for Python variables. Note that Rita was flexibly able to apply mathematical thinking to these variables nonetheless: she switched from Python syntax to mathematical syntax after the first line.)

Their next task was to use the representation of real numbers on the computer to make their own log function. Rita quickly thought of applying logarithm rules to the exponential representation of a number, and wrote down the expression that she had suggested on the whiteboard (Fig. 4). She then asked the interviewer if that was what they were supposed to find. The interviewer confirmed this, and we note that being able to self-assess the results would have been advantageous for the students at this point.

Rita’s further speculation that the point of the exercise was to investigate rounding error suggests that the purpose of manipulating the expression in Fig. 4 was not entirely clear to the students. Even though they were able to do what the task asked them to, they were not sure that they were finished with the task, and we think it likely that they were trying to connect the task to things they had seen in lectures. While Rita’s analysis of the error could be the start of a fruitful mathematical argument in itself, it appears that the purpose of this particular task should have been clarified for the students; in other words, they were not entirely sure where the tutorial was headed and why.

Afterwards, Rita and Lena attempted to connect their work on the whiteboard to what they had learned in class and to the plot from their code, until the interviewer encouraged them to move on to the next task. They went on to write the code for the logarithm function, but they ran into an issue with the provided *taylor* function that calculated values of the Taylor expansion: Rita did not understand why they had to use it and asked the interviewer why that was.

It took only the mention of taylor being an approximation in order to have Rita conclude, correctly, that it was an approximation for the logarithm used in Fig. 4. Until the interviewer mentioned this, she only referred to this function as the sum of general Taylor terms, not something related to the logarithm in particular. Furthermore, she was able to explain the relation between this function, the remainder and the log function the students were tasked to make.

There may be a connection between the tables of variables and functions in the beginning of the tutorial, and this kind of reasoning. Overall, we noted that the students had far less difficulty keeping track of the various variables and functions than the students in the first implementation phase, and we attribute this to these tables and better alignment with the course compendium’s (Mørken, 2017) choices of variable names.

**Fig. 5:**
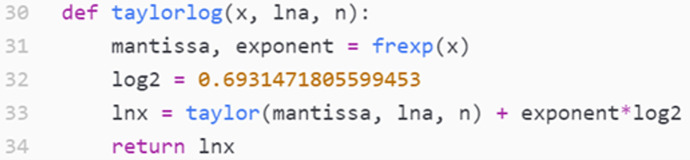
Rita and Lena’s implementation of the logarithm function(*They wrote line 33 and 34; the rest were given in advance.*)

**Fig. 6:**
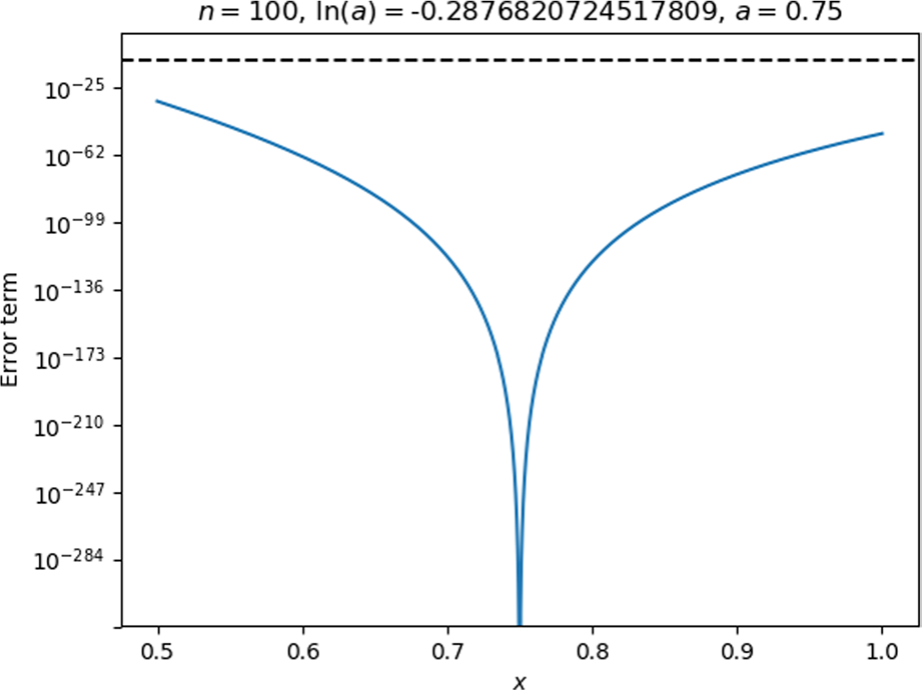
The remainder plot with Rita and Lena’s initial choice of parameters

Rita and Lena wrote the function in Fig. 5, after some further clarifications from the interviewer. They tested the function and confirmed that it produced the expected result. Next, they proceeded to choose better parameters than the default ones. After some discussion and experimentation with parameters, they found out that $n=1000$ made the code run rather slowly. They settled for $n=100$ and decided that they wanted the parameter $a$ in the middle of the interval of interest:

For practical reasons (see Appendix 2), the code used $\mathrm{ln}(a)$ as an input parameter instead of $a$ itself. To get $a$ in the middle of the interval $0.5\le x<1$, the students would need to know the value of $\mathrm{ln}(0.75).$ They could have found one by trial and error through plotting the remainder (as plots like the one in Fig. 6 do display the value of $a$), but Rita expressed some dissatisfaction with this approach, and stated at the end of the interview that she generally liked precise expressions for quantities.

### How Many Terms? A Mathematical Approach

Rita and Lena tested their choice of parameters and found that they resulted in accuracy well beyond what was needed for the entire interval, as seen in Fig. 6. The interviewer, noticing that the accuracy was, in fact, well beyond what the tutorial required,^7^ challenged the students to find out how few terms they could get away with.

They looked up the mathematical formula for the remainder once more and decided to look for an analytical solution. This was not anticipated: the design had simply assumed that the students would use trial and error and let Python do the heavy lifting (which would indeed be a black box approach). That the students themselves saw a possibility we missed as designers was both a pleasant surprise and a reminder that we should not underestimate our students’ potential appreciation of mathematical rigour.

This impromptu addition to the tutorial (that was later added to the final version we describe in our penultimate section) caught the attention of both students, which certainly fits well with the WHERETO prompt for designing learning activities in the UbD framework. In terms of evidence of understanding, we shall soon see that this challenge got them to apply their mathematical knowledge to the task. Beyond this, there are two features of the design that we claim helped the students in this work and may also have influenced them in believing that such an approach would be fruitful in the first place.

First, we see that the students’ attention was directed to the formula, which provided them with a solution for the remainder in the particular case of the logarithm. Having this formula available, whether it be the result of the students’ own work or, as here as a provided resource, may have served as a natural entry point. This formula became the right-hand side of the equation they were about to set up.

Second, we also see that they were given a simple, if arbitrary, threshold for the remainder at 10^−10^, as opposed to not knowing or having to guess. Trusting that this threshold would produce good-enough approximations (which we shall see that it did), supported setting up the left-hand side of the equation. Put more concisely, the design offered up quantities that the students were able to assume being equal to each other, and then investigate analytically what followed from that assumption.

Shortly after asking Lena for the remainder formula of the logarithm (provided in the hand-out we gave the students), Rita repeated her suggestion of setting up an equation, but seemed a little bit unsure whether or not that was better than trial and error. The interviewer commented that if they did not use trial and error, they would need to test for a lot of $x$ values to ensure that the remainder was below the threshold *everywhere*. Rita then demonstrated her understanding by being able to justify the analytical approach:

*Rita: *Oh yeah, no, but I thought that we just tested for 0.5 and 1 because that’s the worst case*.*

*Interviewer: *You wanted to test the end-points where, sort of, it’s the worst case?

*Rita: *Yeah.

*Interviewer: *That sounds like a really good idea to me.

We interpret this exchange to be afforded by two design features. First, the tutorial explicitly mentioned and explained worst-case thinking, in helping students pick a value for the unknown parameter $\xi$ (described in Appendix 2). This might have influenced Rita’s thinking about the current task, as she used the term “worst case” explicitly. Note that Rita provided evidence for perspective here, as she looked at a problem designed to be solved using computing in a mathematical way.

Second, just prior to asking for the formula, Rita had seen the plot of the remainder for the entire interval and noticed its U-shape (Fig. 6) and seemed to have interpreted that “higher is worse” in that plot, allowing her to identify the points that were worse in terms of accuracy than all the rest. In addition to this plot, the students were attending to the example plot in the tutorial (Fig. 13, in Appendix 2) and had both plots side by side on the computer screen.

**Fig. 7:**
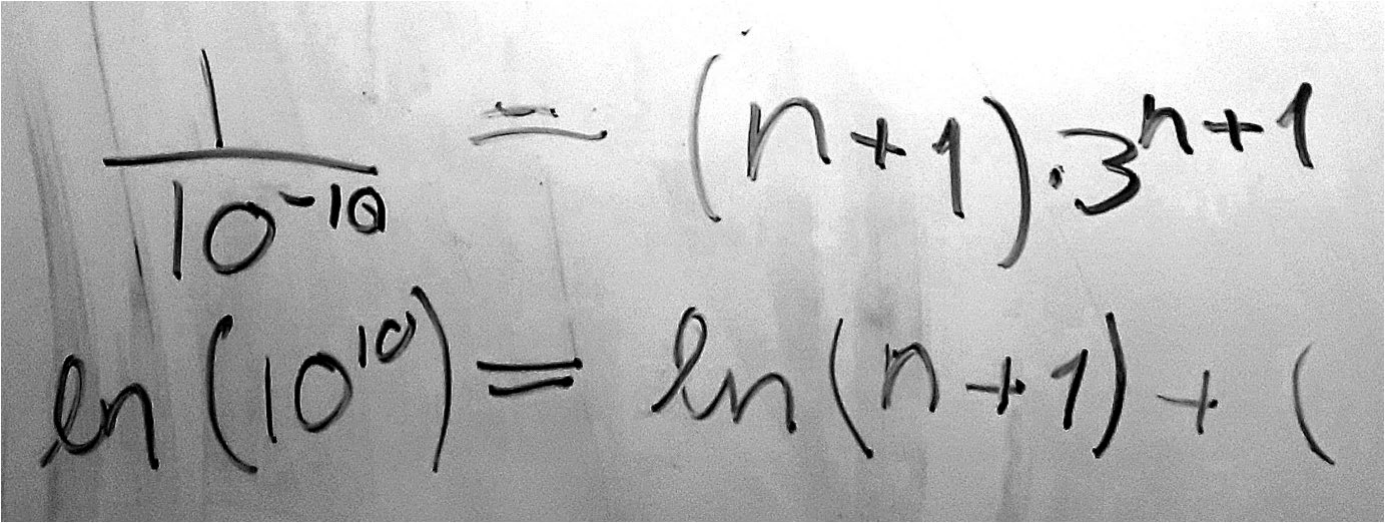
The end-point of Rita’s first whiteboard calculation of the number of terms

Rita attended to the remainder formula and proceeded to set it equal to 10^−10^. She then attempted to solve the resulting equation on the whiteboard. Unfortunately, the unknown parameter $n$ ended up in two different places, as seen in Fig. 7, and the students quite correctly concluded that they lacked the mathematical tools to eliminate one of these while preserving the expression’s correctness. They tried the common approach of taking the logarithm of both sides to get the unknown quantity out of the exponent, but, as a result, ended up trapping the other instance of the unknown inside the logarithm:

The typical way to deal with the latter would be to reverse the process, in effect making it circular without bringing the students closer to a solution. At this point, Lena suggested representing the result of their mathematical work in Python, so that they could find a solution. The students were able to justify why they would want to do that: isolating the unknown variable proved impossible with the methods available to them. Being able to see the problem from the vantage points both of mathematics and of computing fits well with the perspective sub-category of understanding in the UbD framework.

We attribute this particular occurrence to the overall framing of the tutorial as a computational activity. We emphasised the computational nature of the work in the design and provided mathematical resources as handouts separate from the main tutorial. We believe this led the students to expect needing to use Python throughout and to have a low threshold for resorting to computational solutions.

In the same way, it is possible that having many more years of experience with mathematics (as opposed to computing) may have equipped these students with a low threshold for resorting to analytical mathematics on the whiteboard. However, it should be pointed out that giving the students designated responsibilities — one being in charge of the computer, the other the whiteboard — may have enabled each of them look for opportunities to use “their” tool. If so, it may warrant consideration to make this a permanent feature of the tutorials even after COVID-19 safeguards are no longer required.

#### Solving the ‘Unsolvable’ Equation

Rita and Lena’s first attempt at solving the equation using computing was an example of the black-box mentality that we hoped the tutorial would help the students grow out of: Rita asked twice if she could use *GeoGebra* to solve this equation, claiming that would be the simplest way to find the answer (as you could just write the expression) and that she was skilled in this kind of solution. What triggered the black-box approach seemed to be that the students recognised that they were unable to find an analytical solution, by attending to the impossibility of isolating the unknown quantity.

*GeoGebra*, which featured among the examples in the integrated design literature section of this article, is a plotting software which has been commonly used in Norwegian high schools throughout the 2010s. The approach that Rita described would entail simply writing down each side of the equation and reading off the solution based on the point of intersection, and it is possible that this was Rita’s go-to strategy when faced with something she could not solve by ordinary means.

It is important to note that Rita’s explanation is correct: this equation is impossible to solve analytically. Its solution is called the product log function or Lambert W-function, and is simply defined as the inverse function of $f\left(W\right)=W{e}^{W}$ (Weisstein, n.d.). This is not an explicit definition of the kind these students are used to, and we suspect they would not find it very helpful.

While recognising that the equation could not be solved analytically, and that a numerical solution might still be possible demonstrates understanding as in the six facets defined by Wiggins and McTighe, Rita appeared animated when explaining this, and we interpret this as frustration that her mathematical work appeared to be going in circles. She stated in the follow-up questions that she did “like having an expression for things” and seemed to prefer solutions with mathematical rigour. We interpret her preoccupation with using *GeoGebra* to solve the equation as the only alternative she saw when the equation could not be solved by ordinary means.

Lena’s comments, in contrast, seemed to indicate that a solution might be found by hand, but that she doubted she had the required skill. One could argue that *GeoGebra* does not *have* to be used in a black-box fashion, as the students could have discussed the merits of plotting the solution and found an intersection point more generally, perhaps by using Python to do so, since *GeoGebra* was not available during the interview. This they did not do and, alongside Rita’s statement that “GeoGebra can do it for us,” Lena provided support for our interpretation of this event as black-box thinking in the follow-up questions.

After the students had completed the tutorial, she linked using computing in mathematics to convenience:

*Lena: No, really, the thing is that when there are very complicated expressions, or like when we have something raised to the power of a thousand or something like that.*

*Interviewer: Mmm. [affirmative]*

*Lena: Then you do quickly think that it can’t be done by hand. But that it’s quite easy to type. And then you get an answer. So, then you save some time.*

Their first Python attempt went along similar lines, as they translated the equation into their Python editor and tried to find some function that would solve it for them:

*Rita: *How do you, like, get that to solve for $n$*?*

*Lena: *Yeah. Are there some functions in Python? “Solve equation” or something like that?

*Rita: *Maybe. Try some thing or other. Just write something like “from math import *”, it’s guaranteed to be a, sort of, math module.

*Interviewer: *[laughs] I think it’s a bit advanced to do that … to solve it symbolically, that is.

*Lena: *OK.

*Rita:* There isn’t something called “solve”?^8^

The interviewer instead suggested a more low-level approach that (a) leveraged Python features the students had already used in class and (b) focused on *how* Python found the answer rather than the efficiency of finding it. To avoid blatant cues, he described what he wanted the program to do, in hopes that the students would recognise it, rather than using familiar terms. Framing it as having Python try many different values, the students immediately remembered the concept of loops, and the interviewer further suggested that such a loop could run until they were happy with the error.

This exchange demonstrates Rita and Lena’s understanding of a loop as a way for the computer to repeat something with variation. Here, the students showed that they were able to see the challenge of solving the equation from a computational perspective once the relevant concept had been made relevant (and not in an explicit way).

We note that, unlike her statement that she was skilled in *GeoGebra*, Rita stated that she was initially unsure how to implement a similar statement in Python, even after they had translated the idea of a numerical solution to a loop. When the interviewer built on their suggestion by mentioning a *while* loop in particular, and by pointing their attention to the fact that they could decide when the loop should stop, Rita was able to convert her whiteboard equation to a conditional statement that helped her decide when the loop would finish.

Even though conditional statements are written so that the loop continues as long as it evaluates to True, it is often useful to think about it inversely as a stopping condition: we want the loop to stop when we are satisfied with the remainder. Hence, we want the expression to evaluate to False at that point, and not before. Thus, the conditional we desire is one that evaluates to True as long as the remainder remains too large. While the interviewer might have to take the credit for highlighting this idea, attending to the interviewer’s hints and the equation on the whiteboard led Rita and Lena to demonstrate their ability to interpret the situation and apply their programming knowledge by translating the equation into an inequality, which they did of their own accord.

**Fig. 8:**
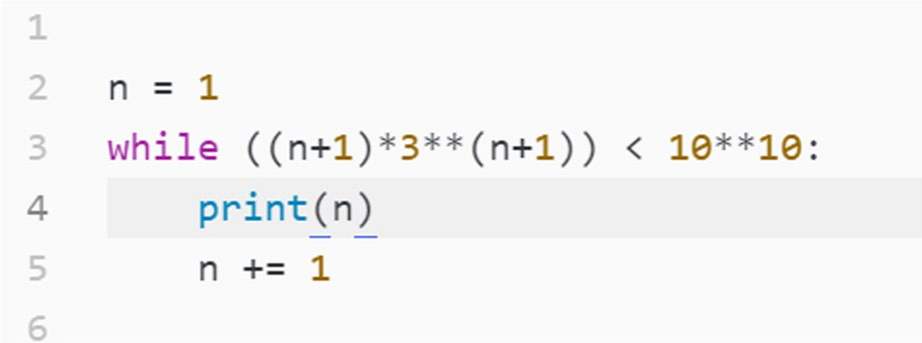
Rita and Lena’s code to solve the equation computationally

**Fig. 9:**
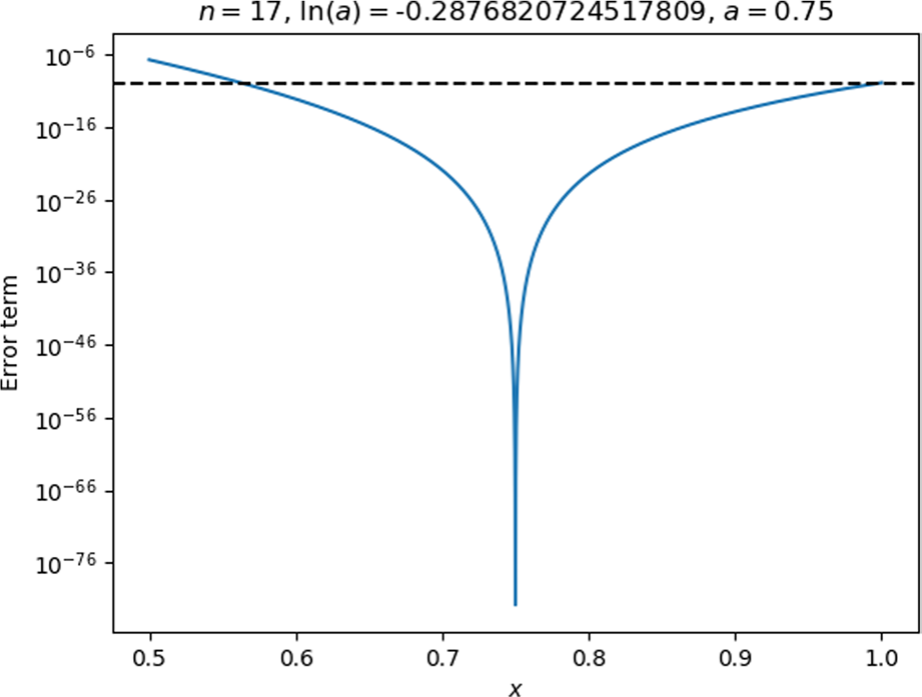
Rita and Lena’s revised parameter choices, leading to a remainder that was too large near $x=0.5$

The students wrote the short program in Fig. 8, containing a *while* loop that would start at the smallest possible number of terms $n=1$ and increase the number of terms^9^ until the accuracy was acceptable. This program used Rita’s whiteboard work as its basis and, unlike the original formula, contained no fractions, negative exponents or absolute values, thereby simplifying the expression considerably. Running their code, Rita and Lena got the results shown in Fig. 9.

While the accuracy was good at $x=1$, the remainder was still too large around $x=0.5$, and the students seemed disappointed with the result until the interviewer offered some encouragement and pointed out that one of the end-points looked good. Rita eagerly responded that she now realised they had to check *both* end-points and pick the largest number of terms that resulted from either of these. This indicates that she not only was able to interpret the output in the context of her earlier whiteboard calculations, but was also able to look forward and apply that interpretation to formulate a plan for getting the result they wanted. It seems that the appearance of the threshold in the plot as a horizontal dashed line was helpful for Rita to come to this conclusion.

Rita then repeated her calculation for $x=0.5$ and, during the process, explained why a smaller value of $x$ led to a larger remainder. While demonstrating that she was able to interpret and explain the situation in this way, Rita was attending to the plot of the remainder (on screen), her earlier equation for $x=1$ (still visible on the whiteboard) and the interviewer’s reminder about the parameter $\xi$ (see Appendix 2) in that equation. Additionally, she took a mathematical perspective to the plot in question.

Rita continued until she arrived at an expression containing a negative sign inside an absolute value, which Lena suggested would in effect cancel out the minus sign. This demonstrates one additional benefit of implementing her work on the whiteboard. Instead of having to use a Python function such as *abs*(…) to deal with the absolute value, they were able to apply their mathematical knowledge to simplify the task and write more readable code. As before, in their finished expression that went into the *while* loop

$$\left(n+1\right)\cdot {2}^{n+1}<{10}^{10}$$

they had removed all fractions and negative exponents from the equation.

While modifying the program, the students first attended to Rita’s whiteboard work (repeated at the interviewer’s request for $x=0.5$) and then their old code for $x=1$. In the end, all that was required was replacing the value 3 with the value 2 (see Table 1).

**Table 1:** Effects of changing $x$ on $\xi$ and the remainder

$x$	$a$	$\xi$	Remainder
1	0.75	0.75	$\frac{1}{n+1}\left|{\left(\frac{1}{3}\right)}^{n+1}\right|$
0.5	0.75	0.5	$\frac{1}{n+1}\left|{\left(-\frac{1}{2}\right)}^{n+1}\right|$

This may look like black-box thinking at first glance. However, we conjecture that this insight built on the mathematical understanding that the students demonstrated earlier, even though they did not repeat these demonstrations at this stage.

If the problem had been framed as a code problem, it is possible that the students would have accepted more convoluted code, as opposed to the observed simplifying what they could before coding. We therefore postulate that having the students take the analytical path as far as they can, before being forced to switch to Python, is desirable in light of the learning goal that students should be able to see problems from both a computational and a mathematical perspective.

This raises an interesting question, however. Is such work devalued (in the students’ eyes) by the existence of a way to get around the problem by using various Python functions and packages instead of mathematics? Rita and Lena’s initial desire to use *math.solve*() and find a quick solution seems to point *away* from the understandings we seek. If the students see computing primarily as a means to make laborious mathematics more convenient, then it is possible that they would prefer the black-box solution.

Their satisfaction with *how* they found their answers might suggest otherwise, though. Rita was able to explain, with some enthusiasm, why the remainder was different between the two end-points, which we posit she could not have done had they simply translated the formula verbatim into Python. It is possible, however, that these students were somewhat exceptional in their appreciation of mathematical rigour. Rita stated during the follow-up questions that she felt her work was “more correct” when she did analytical mathematics by hand, which seems to suggest that she would prefer going as far as possible with math alone before using computational tools.

**Fig. 10:**
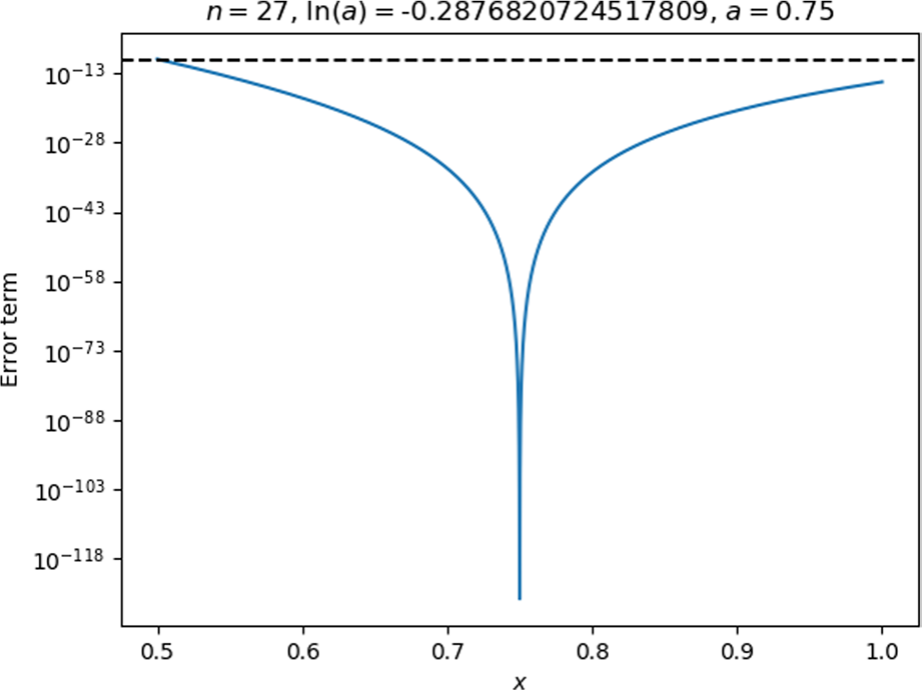
Rita and Lena’s final, optimal choice of parameters

**Fig. 11:**
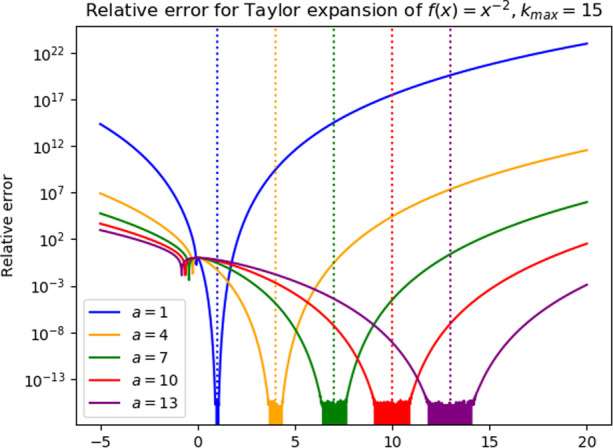
Relative error for the Taylor expansion of $f\left(x\right)={x}^{-2}$, with $n=15$, for different values of $a$ (Originally, we called the number of terms ${k}_{max}$, which confused some of the students, who were used to $n$ from the lectures. As a result, we changed this in the second design phase. Note that the second axis is logarithmic, hence the “noise” for small values of relative error represents the presence of rounding errors. In this plot, we see indications of higher values of $a,$ resulting in a broader region of high accuracy for this function.)

**Fig. 13:**
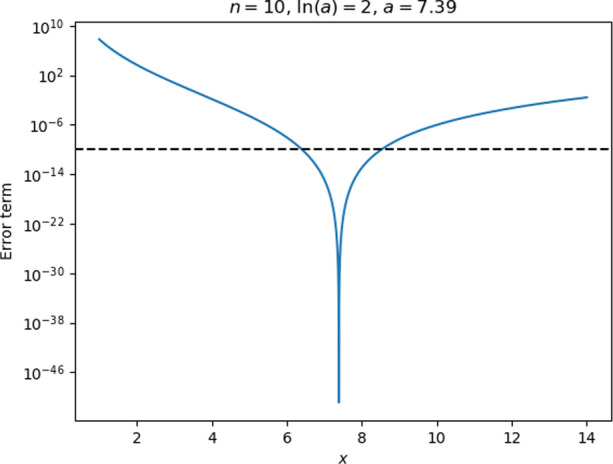
Test case for the remainder function (If the students’ function reproduced this plot, they were free to move on to the next task. Note the slight asymmetry of the curve. The dashed line represents our good-enough threshold value of ${10}^{-10}$ for the remainder. In this second version, the students had to change the plotted region as well, from (1, 14) to (0.5, 1). In the final version of the tutorial, we give them instead an example in the interesting region (0.5, 1) right off the bat, to give students more time to focus on activities that point toward the learning goals.)

After some more work, Rita and Lena found a solution that gave an acceptable remainder for both end-points ($n=27$), see Fig. 10.

The interviewer then suggested they try out their log function from the previous task with these parameters, at which point Rita started considering how they could have found the parameter value $\mathrm{ln}(0.75)$ without resorting to *numpy*’s log function:

*Interviewer: *We did cheese this one^10^a bit, but …

*Lena: *Well, so it was twenty-seven, and seventeen for the last one?

*Rita: *But log nought point seven five, isn’t that … no. Kidding. It’s the logarithm of three over four, that. But it isn’t any … Because we have … we do have the logarithm of a quarter … since that is two times the logarithm of two, but we don’t have the logarithm of three.

*Lena: *No.

*Interviewer: *That would be creative too, I didn’t even think of that.

Here, Rita demonstrated understanding by explaining how to apply the logarithm rules and what information they would need, that is, $\mathrm{ln}(3)$, to find the number they wanted. This reasoning seems to have been prompted by Lena’s question and the interviewer’s sentiment that this was, in a sense, cheating or “cheesing”. Her attempt to find this number by other means can be interpreted as a desire to re-create the work of the original log-function designers in a self-sufficient way, without having a previous log function available.

### Summary

To summarise, we found that quite a few features of the new version aligned with the design standards of the UbD framework, which we used to evaluate the teaching designs. These features are listed in Table 2:

**Table 2:** Alignment of second version features with UbD design standards

Feature	Alignment with UbD standards
Redesigning log function from scratch	Clear goal, hook students’ attention (WHERETO), focus on big ideas
Task goals	Explain, interpret and apply (six facets of understanding)
List of variables with familiar names	Organise for engagement and effectiveness (WHERETO)
On-the-fly suggestion to find *optimal* number of terms	Students need to rethink and revise (WHERETO)
Opportunity for mathematically comparing the remainders at the end-points	Explaining why they are different (six facets of understanding)

However, there remained some issues that we flagged to be of interest for the final design phase. Table 3 summarises these issues.

**Table 3:** Unresolved issues for the final design phase

Issues of interest for the final design phase
Students spent considerably more than the allocated 1 hour on the tutorial
The purpose of the transformation $\mathrm{ln}(M)+E\cdot \mathrm{ln}(2)$ was not sufficiently clear — how may the students evaluate their work on this transformation as sufficient and move on?
The role of the computational function **taylor**() was not sufficiently clear
Using $\mathrm{ln}(a)$ as an input parameter encourages using the optimal value $\mathrm{ln}(0.75)$, but the tutorial does not support finding this value in any meaningful way
Some scaffolding may be needed to break the apparent habit of students looking for black-box solutions to problems they regard as difficult
Will students see the value of the mathematical work when they can also use Python to ‘brute force’ the problem without opportunities to demonstrate understanding?

With these lessons learned in the second round of interviews, we moved to the final design phase.

## Final Design

The first major change we made to the final phase was to make different versions of it to allow for flexibility with regard to time. We initially designed the tutorial to take students one hour to complete, but in practice, the time was closer to one-and-a-half hours, in some cases even two. Nonetheless, our data indicated that the time was well spent by the students. For this reason, we decided to provide two versions of the tutorial that educators could choose from: an extended 2-hour version and the original 1-hour one. In the latter case, we cut some tasks from the tutorial, at the cost of less opportunity for insight and discussion.

In Table 4, we show which tasks are present in each version of the final tutorial, as well as the version that was used in the case that we presented in the previous section. In addition, we suggest an optional exercise that can be used to extend the two-hour version further if desired. These task numbers refer to the final version of the tutorial, published in Appendix B of Sand (2021).

**Table 4:** Versions of the tutorial (in the 1-hour version, students will have access to all the results they need from the tasks that have been omitted). For the interview, Task 1 was omitted due to time constraints, whereas task 8 was not conceived until after the interview, in part based on the interview data. Tasks originated during the interview and which the students completed are marked as “Included” in the right-most column, even though they were not prepared ahead of time.

Tasks	1h version	2h version	Interview: Rita and Lena
1	Not included	Included	Not included
2–3	Not included	Included	Included
4–7	Included	Included	Included
8	Not included	Included	Not included
9	Not included	Included	Included
10	Included	Included	Included
11	Not included	(Optional)	Included
12	Included	Included	Included

From the interview with Rita and Lena, we learned that the purpose of the transforming $\mathrm{ln}(x)$ using logarithm rules should be better motivated. The students needed to know when the task was complete and why we were asking them to do it. To that end, we added a question to have the students consider the accuracy of $\mathrm{taylor}(mantissa)$ compared with $\mathrm{taylor}(x)$. The students may not be used to reasoning in this way; hence, some scaffolding is beneficial to make the purpose of the task clear. This also adds an opportunity for self-assessment, so that they can see whether the result of their work is meaningful.

The next task, asking the students to implement their own log function, also needed revision. Rita and Lena had not realised that the function $\mathrm{taylor}$ was meant to be used as a stand-in for the logarithm itself. Therefore, we clarified the text of the task to highlight this fact.

The interviewer’s suggestion, that the students figure out how few terms one could get away with, was met with enthusiasm by Rita and Lena, who used both traditional math and computing to pin-point this number in a flexible way. We therefore added this as a formal task, encouraging students to use traditional math as far as they were able, then switching to Python if need be. We kept the hint asking if there were a way to have Python test many different values of $n$, as that proved effective at getting Rita and Lena to think about loops.

Finding the value of $\mathrm{ln}(0.75)$ to machine accuracy turned out to be reasonably simple using the Taylor expansion with $a=1$:

$$\mathrm{ln}(0.75)\approx \mathrm{ln}(1)+\sum_{i=1}^{n}\frac{{\left(-1\right)}^{i-1}(i-1)!{1}^{-i}}{i!}{\left(0.75-1\right)}^{i}=\sum_{i=1}^{n}-\frac{{0.25}^{i}}{i}$$

The students can use the provided Python function taylor to calculate this. We could instruct them to set $n=100$, which from the experience of Rita and Lena is likely to be more than enough to save them the trouble of estimating the optimal number of terms a second time. This demonstrates that Taylor expansions can also be used to find the values of troublesome constants, and that the computer is well suited to work with these expansions.^11^

Considering the length and complexity of the tutorial overall, however, we decided simply to provide the machine-accurate constant for $\mathrm{ln}(3)$ instead. If the students have time to spare, it is better spent working with the optional task of comparing the two remainders at the end-points and explain why one is smaller than the other. Based on this experience, we believe that asking students to explain this is another way to produce evidence of their understanding.

## Discussion

In this final section, we discuss our findings and relate them to our research questions, before outlining future possibilities for research.

### Design Features

The first research question concerned the features students attended to when demonstrating understanding. While we do not have direct access to students’ thought processes, our analysis identified three core features that they looked at or talked about just before or during processes of explaining, interpreting and applying knowledge. These were (a) the purpose of code, (b) plots, and (c) handwritten math.

The purpose of code can be thought of in terms of its *interface* (the “what”), as opposed to its *implementation* (the “how”). When thinking about the latter, we saw our students engage in black-box thinking, especially if the problem seemed difficult or tedious. It was when they considered the former (as Rita and Lena did with the *taylor* function) that we saw the students demonstrate understanding as defined by the UbD framework. This suggests that students are less likely to slip into black-box thinking when they are encouraged to take the bird’s eye-view, enabling them to see the forest for the trees.

The plots, especially the ones the students had made themselves, and the students’ mathematical work also provided several opportunities for demonstrating understanding. A good example of both is Rita using the plot in Fig. 9 and her on-going whiteboard work to explain why the remainder looked different at the end-points. We find it very interesting that both computational and mathematical representations were used together to demonstrate understanding. This result is similar to the findings of Lockwood and De Chenne (2020), in which students used computational representations (code and output) to explain the connection between mathematical concepts (combinatorial problems and their outcomes). Yet our results are also different, because our students used representations from *both* domains in their mathematical reasoning.

### Mathematical and Computational Concepts

Given our learning goals, the second research question focused on which concepts the students understood. In light of this, we would like to highlight three concepts that our students two sections back demonstrated an understanding of (a) the usefulness of Taylor expansions and the remainder, (b) worst-case thinking, and (c) the relationship between mathematics and computing.

Our first group of students grappled with the questions of what Taylor polynomials are and what they are good for. As we mentioned earlier, this experience is not unique. Rita and Lena, on the other hand, had an epiphany when they considered the *taylor* function as an approximation that allowed the computer to calculate something complex to arbitrary precision, and the remainder as a way of measuring how good this approximation was. This suggests that our approach allowed students to focus on the “why” as opposed only to the “how,” which was the essence of the questions these students were asking.

Rita was also able to generalise the concept of worst-case thinking: she explained that, instead of checking all possible cases, we can check only on the ‘worst’ ones — if they are acceptable, all other cases will be as well. This concept is obviously useful in making analyses of very large data sets tractable and efficient. We posit that employing this kind of mathematical reasoning led the students to write more efficient and readable code, as opposed to a brute-force approach that let the computer do all the heavy lifting. A similar argument can be made for the simplifications the students made to their equation.

This leads us to the third concept: how mathematics and computing relate to each other, as experienced by the students. Rita and Lena were guided by the tutorial to combine logarithm rules and computational representations of floating-point numbers. This resulted in a powerful transformation that mapped a half-open set to a closed one. In this case, we would have liked the students to explain in greater detail what they thought was going on. To what extent they were able to generalise the experience unfortunately remains an open question.

We are better positioned to claim that the students understood that one can use an equation to find the input of a program that produces desired output, and that one may use a loop to solve an equation that is very hard or impossible to solve by hand. In these cases (despite some hints from the interviewer), the students largely came up with these ideas on their own. We cannot claim that this experience led to students being able to generalise these understandings to other contexts, as Wiggins and McTighe (2005) and Lobato (2012) emphasise. We have identified a context, however, in which the students were able to demonstrate these understandings.

### Design Lessons Learned

Our final research question concerns the lessons we, as designers, learned from the process of iterative design. We identified four major take-aways: (a) clarity matters, (b) re-invent something real to engage the students, (c) students need dots to connect, and (d) students need opportunities to self-assess.

One of the major differences between the first two initial designs involves the amount of time students spent understanding the information and the tasks on the worksheets. For instance, much time in the first version was spent remembering what the variables stood for, as some of them were given different names from the ones the students were used to. Collecting all variables and functions in tables with short explanations proved very helpful to students in the second version of the tutorial. Because the students’ time is valuable, and cognitive load is a limiting factor to understanding, clarity matters.

While our sample size is too small to claim generalisability, we have demonstrated the possibility of students engaging strongly with the premise of re-inventing early work whose results they use often. The group of students that struggled with purpose and direction in the first version were excited by the mere idea, and the second version students demonstrated that the work felt relevant and authentic to them, as Wiggins and McTighe (2005) prescribe. Likewise, the air of mystery from the essential question, “How do you calculate logarithms with only the four arithmetic operations?,” sustained students’ engagement throughout. We should note, however, that the research interview setting and access to a “TA” at all times might have contributed to this effect, so this conclusion is not definitive. Nonetheless, our results suggest that mystery and authenticity engaged the students, which makes for effective learning.

From our first version, especially, we learned that if students are to connect the dots and relate pieces of knowledge, they need dots to connect: assuming students have prerequisite knowledge is dangerous, as in the case of dominating factors. Furthermore, even when students have the dots, they may need to see them as relevant in context, as in the case of loops in the context of equations. An important design lesson is therefore that students should have opportunities to review “basic” knowledge and to see this knowledge as relevant and useful, because this helps them connect the knowledge that they have. This supports their demonstrating understanding through applying knowledge, in the terms of Wiggins and McTighe (2005).

Finally, students need opportunities to self-assess, especially in tasks that build on the results of previous tasks. In the first instance, we saw confusion as a result of an early error, which the students had no opportunities to detect until they failed to get the result that the tutorial described in vague terms. While ‘debugging’ both code and mathematical work can sometimes be useful practice, it is generally better to give students test cases early and often, so the search space for errors is limited and they spend their time more productively.

### Future Research

In addition to these findings, we have identified several promising prospects for future research. One could investigate further the split between interface and implementation of functions mentioned under “Design Features” earlier in this section, in order to find out how common it is that students are unable to recognise what a piece of code is doing, because they are too focused on the specific details of how it is done. In computer science education terms, if the students manifest an understanding of all the parts, but not the relations between them, they have difficulties considering a block of code as a whole (Lister et al., 2006). What we would propose, then, is to extend this research to computing in a mathematical context.

Another avenue of future research that we regard as promising is to investigate how widespread black-box thinking is among high-school students, and how the formation of this mindset is influenced by the way computational aids are used in high-school mathematics classes. With the introduction of computer programming into Norwegian school mathematics, students will no longer get a fresh start with computing when they enter university and, as such, a comparative study between current and future students might also be possible for a limited time.

To comply with COVID-19 protocols and recommendations, we gave each student in the second phase groups a designated responsibility: one worked on the computer, as Lena did, while the other wrote on the whiteboard, like Rita. It is possible that having each student represent a particular perspective in this way was beneficial to the students, and we cannot rule out that some of the results were affected by this designation. While we also saw examples of Rita using computational language, and Lena mathematical, we see value in investigating the effects of this way of working in a separate follow-up study, either to strengthen the claims in this article or to identify another potentially important design factor.

Finally, we envision studies involving a larger number of students. One way to accomplish this is to put the finished tutorials in a format that allows for assessing evidence of understanding more systematically, building on the UbD framework and the findings of this article. This would allow for investigation of how widespread these understandings are in the classroom, and how they are distributed among the student population. This would necessitate the design of rubrics to assess to what extends the students understand in different ways, which is something the UbD framework already supports.

## Figures and Tables

**Fig. 12:**
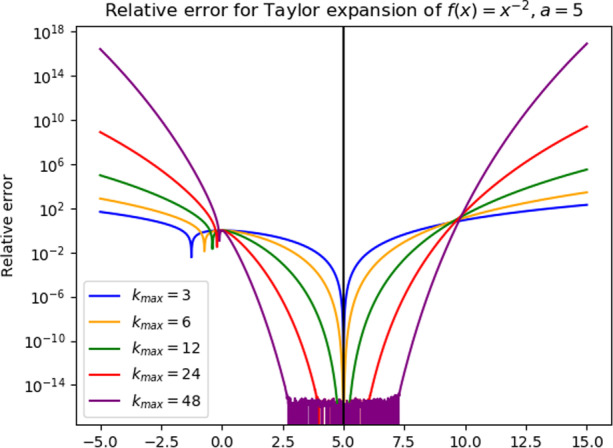
Relative error for the Taylor expansion of $f\left(x\right)={x}^{-2}$, with $a=5$, for different numbers of terms (note the divergence outside the interval $0<x<2a$)

## Appendix

### Appendix 1. Initial tutorial design details

Earlier, we summarised a peculiar feature of the logarithm and its derivatives, namely that, outside a certain region of convergence, adding more terms to the Taylor polynomial makes the accuracy progressively *worse.* This has profound consequences for our choice of the point $a$ used to create the Taylor polynomial: for this class of functions, the choice of $a$ cannot simply be dictated by convenience, but must be chosen so that for a maximal input to the function ${x}_{max}$ we want to Taylor expand around the point $a={x}_{\mathrm{max}}/2$ (see Example 9.10 in Mørken, 2017).

As seen from the remainder when plotted (see Fig. 6), it is desirable to have $a$ in the middle of the interval. Then, all that remains is to determine the number of terms $n$ one needs to ensure the required accuracy. For this, one needs only to consider the worst-case values $x=0$ and $x={x}_{\mathrm{max}}$, since the error (the remainder) grows smaller the closer to $a$ one comes.

After the initial task, we asked our students to find the Taylor expansion of $f\left(x\right)={x}^{-2}$, providing the first few derivatives to enable them to see the general pattern ${f}^{\left(i\right)}\left(a\right)={\left(-1\right)}^{i+1}\left(i+1\right)!{a}^{-(2+i)}$ that they could plug into the Taylor formula. Next, we asked them to implement the following mathematical functions as Python functions (here presented with correct solutions):

$$\begin{array}{c}\mathrm{taylorterm}\left(x,a,i\right)=-\frac{\left(i+1\right)}{{a}^{2}}{\left(1-\frac{x}{a}\right)}^{i}\\ \mathrm{relerr}\left(y, {y}_{\mathrm{exact}}\right)=\left|\frac{y-{y}_{\mathrm{exact}}}{{y}_{\mathrm{exact}}}\right|\end{array}$$

The first function returns term number $i$ of the Taylor polynomial^12^ and the latter is the relative error, a common measure of accuracy. These functions would then be used by two programs that we provided the students with. These programs plotted the relative error as a function of $x$ in two different ways: (a) for several different values of $a$, keeping $n$ constant (Fig. 11) and (b) for several different values of $n$, keeping $a$ constant (Fig. 11).

After producing the plots, the students were allowed to change the constant parameter of each program and asked to explain the effects of both parameters. We asked them to interpret what the plots were telling us, especially concerning the rounding errors in the bottom of the plots, and the regions evident in Fig. 12, where more terms make the approximation worse.

### Appendix 2. Second tutorial design details

Earlier, we broadly described how computers implement logarithm functions and highlighted the importance of mapping all positive real numbers to a small interval, so that one can control the accuracy. One way to do this mapping is to exploit the way a positive real number is represented as a mantissa and an exponent in the computer (which, incidentally, was already part of the course curriculum),

$$x=M\cdot {2}^{E},$$

where $0.5\le M<1$ and $E$ is an integer. We can obtain $M$ and $E$ using the Python function called math.frexp(), which the students used in Fig. 5. Taking the logarithm of each side and applying logarithmic laws with which the students are familiar, we obtain

$$\mathrm{ln}(x)=\mathrm{ln}(M)+E\cdot \mathrm{ln}(2)$$

which simplifies things more than is apparent at first glance. If we use Taylor polynomials for our logarithms,^13^$\mathrm{ln}(x)$ requires machine-accuracy for all real numbers, whereas $\mathrm{ln}(2)$ is a known constant, and $\mathrm{ln}(M)$ only requires us to approximate the logarithm accurately for numbers between 0.5 and 1 (Hammen, 2012).

In the course compendium, the absolute value of the remainder can be written in two ways. The first involves an integral, and we judged that adding a second layer of inaccuracy by having the student integrating numerically was needlessly complex. Therefore, we chose the other representation of the remainder of a Taylor polynomial $n$ terms:

$$R\left(x, a, n\right)=\left|\frac{{f}^{\left(n+1\right)}\left(\xi \right)}{\left(n+1\right)!}{\left(x-a\right)}^{n+1}\right|.$$

In fact, the only thing distinguishing this expression from that of the $(n+1)$ term of the same Taylor polynomial is the replacement of ${f}^{\left(n+1\right)}(a)$ by ${f}^{\left(n+1\right)}(\xi )$, where $\mathrm{min}\left(a, x\right)\le \xi \le \mathrm{max}(a,x)$. We do not know the precise value of this number unless $x=a$, in which case the remainder is simply zero (Mørken, 2017, pp. 220–221).

We decided that we could use this limitation to make a point to the students: when in doubt, pick the worst-case scenario. In this case, that meant choosing the $\xi$ that makes the remainder as large as possible. That way, we do not risk underestimating the error, only overestimating it. In our case, $f\left(x\right)=\mathrm{ln}(x)$, the absolute remainder becomes:

$$R\left(x, a, n\right)=\left|\frac{1}{n+1}{\left(\frac{x-a}{\xi }\right)}^{n+1}\right|.$$

In this case, with $\xi$ in the denominator, we obtain the largest possible remainder by picking $\xi =\mathrm{min}(x,a)$. The resulting expression would be usable by the students to plot the accuracy of their approximation over a range of $x$ values, without resorting to a direct comparison with a pre-existing log function, as in the relative error approach we described above in Appendix 1.

To ensure that students got the plots that we expected them to, we gave them a test case using some default parameters to test and self-asses their function with (see Fig. 13). Note that we used $\mathrm{ln}(a)$ instead of $a$ as a parameter for these functions, so that the zero term of the Taylor polynomial would simply be the value of that parameter. We can always recover $a={e}^{\mathrm{ln}(a)}$, and this approach ensures that we do not have to calculate $\mathrm{ln}(a)$ — we can simply *choose* it instead.

## Footnotes

1 First, one considers the big picture (the subject of the activity taken as a whole), then one goes into the details and, finally, one goes back to the big picture and connect the dots between those detailed pieces of knowledge.

2 See footnote 8 for a good example of something the students attempted that we later confirmed would have cost them a lot of time with little to show for it.

3 The exception to this is that we also identified black-box thinking, which is not covered by Wiggins and McTighe. On these occasions, we looked for instances in which the students did not display understanding and, at the same time, when they attempted to use the computer to find an answer for them without engaging with how the computer did that or how one may be sure that one can trust this answer. In short, these are instances in which the students treated the computer as an expert.

4 The error where he had ${f}^{\left(i\right)}(x)$ instead of ${f}^{\left(i\right)}(a)$ was still present in Martin’s expression, but that, in itself, would not have prevented them from completing this particular task.

5 An example of such a comparison when $i$ grows large would be that $i!>{c}^{i}>{i}^{m}$, where $c>1$ is a real number and $m>1$ is an integer. In short, factorials dwarf exponentials, which again dwarf integer powers.

6 The narrow area of convergence: $0<x<2a$.

7 A remainder smaller than 10^−30^, or 30 decimals would be much more accurate than the 15–16 decimals the computer can accurately represent in 64-bit double precision. Hence, the rounding error that comes from using this precision would be about 10^15^ times larger than the mathematical error in the Taylor expansion itself.

8 Actually, there is! After the interview, we used sympy to see what such an attempt might have looked like: import sympy n = sympy.Symbol(‘n ‘) sympy.solve((n + 1)*3**(n + 1) – 10**10, n) This code, however, proved to be very resource-intensive and ran for fifteen minutes before it was terminated without producing any result. We take this as additional justification for directing the students away from this path.

9 The interviewer had not suggested that the loop ran through values of $n$ in ascending order; this was the students’ own idea. It might be that the interviewer’s suggestion of the loop stopping when the remainder was good enough was sufficient to have the students start with as few terms as possible and gradually move on to a larger number of terms, though they did not articulate their reasons for writing the program in this way.

10 The interviewer allowed the calculation of $\mathrm{ln}(0.75)$ using the built-in Python function to save time, after the students had expressed a preference for having $a$ in the middle of the interval.

11 Alternatively, one can write a Python program that looks for a fraction $-\frac{n}{d}$ that is as close as possible to $\mathrm{ln}(0.75)$ as possible. By starting with $n=1$ and $d=4$, incrementing both by one whenever $-\frac{n}{d}>\mathrm{ln}(0.75),$ or incrementing $d$ by one otherwise, we found that $-21/73$ is a very good approximation. The downside of this approach is that we needed an accurate log function to estimate the error for each fraction, which is not optimal if the context is the pretence that no such function exists, and we are trying to write the first one.

## References

[CR1] Ambrose, B. (2004). Investigating student understanding in intermediate mechanics: Identifying the need for a tutorial approach to instruction. *American Journal of Physics,**72*(4), 453–459.

[CR2] Arlin, K. (2012). *What are the practical applications of the Taylor Series?* Stack Exchange. (https://math.stackexchange.com/questions/218421/what-are-the-practical-applications-of-the-taylor-series) Accessed 13 Jan 2021.

[CR3] Ben-El-Mechaiekh, H., Buteau, C., & Ralph, W. (2007). MICA: A novel direction in undergraduate mathematics teaching. *Education Notes,**39*(6), 9–11. (https://brocku.ca/mathematics/resources/learningtools/learningobjects/Notesv39n6pp9-11.pdf) Accessed 2/4/2021.

[CR4] Benton, L., Hoyles, C., Kalas, I., & Noss, R. (2017). Bridging primary programming and mathematics: Some findings of design research in England. *Digital Experiences in Mathematics Education,**3*(2), 115–138.

[CR5] Benton, L., Saunders, P., Kalas, I., Hoyles, C., & Noss, R. (2018). Designing for learning mathematics through programming: A case study of pupils engaging with place value. *International Journal of Child-Computer Interaction,**16*, 68–76.

[CR6] Billett, S. (2013). Recasting transfer as a socio-personal process of adaptable learning. *Educational Research Review,**8*, 5–13.

[CR7] Buteau, C., & Muller, E. (2017). Assessment in undergraduate programming-based mathematics courses. *Digital Experiences in Mathematics Education,**3*(2), 97–114.

[CR8] Buteau, C., Gueudet, G., Muller, E., Mgombelo, J., & Sacristán, A. (2020). University students turning computer programming into an instrument for ‘authentic’ mathematical work. *International Journal of Mathematical Education in Science and Technology,**51*(7), 1020–1041.

[CR9] Caballero, M. (2015). Computation across the curriculum: What skills are needed? (http://arxiv.org/abs/1507.00533) Accessed 1/10/2018.

[CR10] Caballero, M., Chonacky, N., Engelhardt, L., Hilborn, R., del Puerto, M., & Roos, K. (2019). PICUP: A community of teachers integrating computation into undergraduate physics courses. *The Physics Teacher,**57*(6), 397–399.

[CR11] Caballero, M., Wilcox, B., Pepper, R., & Pollock, S. (2013). ACER: A framework on the use of mathematics in upper-division physics. In P. Engelhardt, A. Churukian & S. Rebello (Eds) *Proceedings of the Physics Education Research Conference* (vol. 1513, issue 1, pp. 90–93). College Park, Maryland: American Institute of Physics. (10.1063/1.4789659)

[CR12] Caglayan, G. (2016). Teaching ideas and activities for classroom: Integrating technology into the pedagogy of integral calculus and the approximation of definite integrals. *International Journal of Mathematical Education in Science and Technology,**47*(8), 1261–1279.

[CR13] Cetin, I., & Dubinsky, E. (2017). Reflective abstraction in computational thinking. *The Journal of Mathematical Behavior,**47*, 70–80.

[CR14] DeJarnette, A. (2019). Students’ challenges with symbols and diagrams when using a programming environment in mathematics. *Digital Experiences in Mathematics Education,**5*(1), 36–58.

[CR15] Dimiceli, V., Lang, A., & Locke, L.-A. (2010). Teaching calculus with Wolfram|Alpha. *International Journal of Mathematical Education in Science and Technology,**41*(8), 1061–1071.

[CR16] du Bolay, B., O’Shea, T., & Monk, J. (1981). The black box inside the glass box: Presenting computing concepts to novices. *International Journal of Man-Machine Studies,**14*(3), 237–249.

[CR17] Enelund, M., & Larsson, S. (2006). A computational mathematics education for students of mechanical engineering. *World Transactions on Engineering and Technology Education,**5*(2), 329–332.

[CR18] Enelund, M., Larsson, S., & Malmqvist, J. (2011). Integration of a computational mathematics education in the mechanical engineering curriculum. In P. Hussmann (Ed.), *Proceedings of the 7th International CDIO Conference*, (17). Copenhagen, Denmark: Technical University of Denmark.

[CR19] Falbel, A. (1991). The computer as a convivial tool. In I. Harel & S. Papert (Eds.), *Constructionism* (pp. 29–37). Ablex Publishing.

[CR20] Farris, A., & Sengupta, P. (2014). Perspectival computational thinking for learning physics: A case study of collaborative agent-based modeling. In J. Polman, E. Kyza, D. O’Neill, I. Tabak, W. Penuel, S. Jurow, K. O’Connor, T. Lee & L. D’Amico (Eds), *Proceedings of the 11th International Conference of the Learning Sciences: Learning and Becoming in Practice* (vol. 2, pp. 1102–1106). Boulder, CO: The International Society of the Learning Sciences. (https://www.isls.org/icls/2014/downloads/ICLS%202014%20Volume%202%20(PDF)-wCover.pdf) Accessed 3/24/2022.

[CR21] Farris, A., Dickes, A., & Sengupta, P. (2020). Grounding computational abstractions in scientific experience. In M. Gresalfi & I. Horn (Eds), *Proceedings of the 14th International Conference of the Learning Sciences: The Interdisciplinarity of the Learning Sciences* (pp. 1333–1340). Nashville, TN: The International Society of the Learning Sciences.

[CR22] Feurzeig, W., & Papert, S. (2011). Programming-languages as a conceptual framework for teaching mathematics. *Interactive Learning Environments,**19*(5), 487–501.

[CR23] Gravemeijer, K., Stephan, M., Julie, C., Lin, F.-L., & Ohtani, M. (2017). What mathematics education may prepare students for the society of the future? *International Journal of Science and Mathematics Education,**15*(1), 105–123.

[CR24] Greenstein, S. (2018). Designing a microworld for topological equivalence. *Digital Experiences in Mathematics Education,**4*(1), 1–19.

[CR25] Hambrusch, S., Hoffmann, C., Korb, J., Haugan, M., & Hosking, A. (2009). A multidisciplinary approach towards computational thinking for science majors. *ACM SIGCSE Bulletin,**41*(1), 183–187.

[CR26] Hammen, D. (2012). *How are logarithms programmed?* Stack Overflow. (https://stackoverflow.com/questions/10732034/how-are-logarithms-programmed) Accessed 2/1/2021.

[CR27] Hewitt, D. (2016). Designing educational software: The case of Grid Algebra. *Digital Experiences in Mathematics Education,**2*(2), 167–198.

[CR28] Hoyles, C., & Noss, R. (2015). A computational lens on design research. *ZDM: The International Journal on Mathematics Education*, *47*(6), 1039–1045.

[CR29] Johnson, J. (2011). *The power of Taylor series*. Stack Exchange. (https://math.stackexchange.com/questions/73733/the-power-of-taylor-series) Accessed 1/13/2021.

[CR30] Kirschner, P., Sweller, J., & Clark, R. (2006). Why minimal guidance during instruction does not work: An analysis of the failure of constructivist, discovery, problem-based, experiential, and inquiry-based teaching. *Educational Psychologist,**41*(2), 75–86.

[CR31] Lister, R., Simon, B., Thompson, E., Whalley, J., & Prasad, C. (2006). Not seeing the forest for the trees: Novice programmers and the SOLO taxonomy. In R. Davoli (Ed.), *Proceedings of the 11th Annual SIGCSE Conference on Innovation and Technology in Computer Science Education* (pp. 118–122). New York, NY: Association for Computing Machinery.

[CR32] Lobato, J. (2012). The actor-oriented transfer perspective and its contributions to educational research and practice. *Educational Psychologist,**47*(3), 232–247.

[CR33] Lockwood, E., & De Chenne, A. (2020). Enriching students’ combinatorial reasoning through the use of loops and conditional statements in Python. *International Journal of Research in Undergraduate Mathematics Education,**6*(3), 303–346.

[CR34] Lockwood, E., & De Chenne, A. (2021). Reinforcing key combinatorial ideas in a computational setting: A case of encoding outcomes in computer programming. *The Journal of Mathematical Behavior*, *62*, (#100857).

[CR35] Lockwood, E., & Mørken, K. (2021). A call for research that explores relationships between computing and mathematical thinking and activity in RUME. *International Journal of Research in Undergraduate Mathematics Education,**7*(3), 404–416.

[CR36] Magana, A., Falk, M., & Reese, M. (2013). Introducing discipline-based computing in undergraduate engineering education. *ACM Transactions on Computing Education*, *13*(4), (#16).

[CR37] Malthe-Sørenssen, A., Hjorth-Jensen, M., Langtangen, H., & Mørken, K. (2015). Integrating computation in the teaching of physics. *UNIPED*, *38*(4), 303–310. (http://hplgit.github.io/cse-physics/doc/pub/uniped15.html) Accessed 11/18/2021.

[CR38] Mørken, K. (2017). *Numerical algorithms and digital representation*. Oslo, Norway: University of Oslo. (https://www.uio.no/studier/emner/matnat/math/MAT-INF1100/h17/kompendiet/matinf1100.pdf) Accessed 1/29/2021.

[CR39] Mørken, K. (2021). *MAT-INF1100: Modelling and computations*. Oslo, Norway: University of Oslo. (https://www.uio.no/studier/emner/matnat/math/MAT-INF1100/index-eng.html) Accessed 1/29/2021.

[CR40] Nederbragt, A. (2020). *BIOS1100: Introduction to computational models for Biosciences.* Oslo, Norway: University of Oslo. (https://www.uio.no/studier/emner/matnat/ibv/BIOS1100/index-eng.html) Accessed 2/4/2021.

[CR41] NRC (2012). *Discipline-based education research: Understanding and improving learning in undergraduate science and engineering*. Washington, DC: The National Academies Press. (10.17226/13362)

[CR42] Odden, T., Lockwood, E., & Caballero, M. (2019). Physics computational literacy: An exploratory case study using computational essays. *Physical Review Physics Education Research*, *15*(2), (#020152).

[CR43] Olsson, J. (2019). Relations between task design and students’ utilization of GeoGebra. *Digital Experiences in Mathematics Education,**5*(3), 223–251.

[CR44] Papert, S. (1980/1993). *Mindstorms: Children, computers, and powerful ideas* (2^nd^ edn). New York, NY: Basic Books.

[CR45] Ramler, I., & Chapman, J. (2011). Introducing statistical research to undergraduate mathematical statistics students using the guitar hero video game series. *Journal of Statistics Education*, *19*(3), (22).

[CR46] Redish, J. (2009). *Tutorials from the UMd PERG*. College Park, MD: Physics Education Research Group, University of Maryland. (http://umdperg.pbworks.com/w/page/10511238/Tutorials%20from%20the%20UMd%20PERG) Accessed 1/29/2021.

[CR47] Reeves, T., Herrington, J., & Oliver, R. (2005). Design research: A socially responsible approach to instructional technology research in higher education. *Journal of Computing in Higher Education,**16*(2), 96–115.

[CR48] Sand, O. (2021). *Integrating computing with mathematics and science education: Case studies of student understanding and teaching design*. Unpublished doctoral dissertation. Oslo, Norway: University of Oslo. (https://www.duo.uio.no/handle/10852/88987) Accessed 11/20/2021.

[CR49] Sengupta, P., Dickes, A., & Farris, A. (2018). Toward a phenomenology of computational thinking in STEM education. In M. Khine (Ed.), *Computational thinking in the STEM disciplines: Foundations and research highlights* (pp. 49–72). Springer.

[CR50] Šikić, Z. (1990). Taylor’s theorem. *International Journal of Mathematical Education in Science and Technology,**21*(1), 111–115.

[CR51] Sinclair, N., & Patterson, M. (2018). The dynamic geometrisation of computer programming. *Mathematical Thinking and Learning,**20*(1), 54–74.

[CR52] Teegavarapu, S., Summers, J., & Mocko, G. (2008). Case study method for design research: A justification. In *Proceedings of the ASME International Design Engineering Technical Conference & Computers and Information in Engineering* (vol. 4, pp. 495–503). Brooklyn, NY: The American Society of Mechanical Engineers. (10.1115/DETC2008-49980)

[CR53] van Someren, M., Barnard, Y., & Sandberg, J. (1994). *The think-aloud method: A practical guide to modelling cognitive processes*. Academic Press.

[CR54] Wagh, A., Horn, M., Levy, S., Guo, Y., Brady, C., & Wilensky, U. (2017). Anchor code: Modularity as evidence of conceptual learning and computational practices of students using a code-first environment. In B. Smith, M. Borge, E. Mercier & K. Lim (Eds), *Making a difference: Prioritizing equity and access in CSCL. Proceedings of the 12th International Conference on Computer-Supported Collaborative Learning (CSCL 2017)* (vol. 2, pp. 656–659). Philadelphia, PA: International Society of the Learning Sciences.

[CR55] Watters, D., & Watters, J. (2006). Student understanding of pH: “I don’t know what the log actually is, I only know where the button is on my calculator.” *Biochemistry and Molecular Biology Education,**34*(4), 278–284. 10.1002/bmb.2006.494034042628 21638692

[CR56] Weintrop, D., Beheshti, E., Horn, M., Orton, K., Jona, K., Trouille, L., & Wilensky, U. (2016). Defining computational thinking for mathematics and science classrooms. *Journal of Science Education and Technology,**25*(1), 127–147.

[CR57] Weisstein, E. (n.d.). *Lambert W-Function*. Wolfram Mathworld. Champaign, IL: Wolfram Research, Inc. (https://mathworld.wolfram.com/LambertW-Function.html) Accessed 1/6/2022.

[CR58] Wiggins, G., & McTighe, J. (2005). *Understanding by design* (2nd ed.). Association for Supervision and Curriculum Development.

